# Enhancing Thrombolysis Safety in Post-Acute Ischemic Stroke with Tissue Plasminogen Activator-Associated Microparticles

**DOI:** 10.1021/acsnano.5c01499

**Published:** 2025-06-11

**Authors:** Raffaele Spanò, Corinne Portioli, Tijana Geroski, Alessia Felici, Anna Lisa Palange, Peter James Gawne, Stefania Mamberti, Greta Avancini, Roberto Palomba, Thomas Bonnard, Thomas Lee Moore, Massimo Del Sette, Nenad Filipovic, Denis Vivien, Paolo Decuzzi

**Affiliations:** † Laboratory of Nanotechnology for Precision Medicine, 121451 Fondazione Istituto Italiano di Tecnologia, Genoa 16163, Italy; ‡ 127740 Faculty of Engineering, University of Kragujevac, Kragujevac 34000, Serbia; § Institute Blood and Brain @ Caen-Normandie (BB@C), 551958 Physiopathology and Imaging of Neurological Disorders (PhIND), Normandie University, UNICAEN, INSERM, GIP Cyceron, Caen 14000, France; ∥ Neurology Unit and Stroke Unit, IRCCS Ospedale Policlinico San Martino, Genoa 16132, Italy; ⊥ Clinical Research Department, CHU de Caen Normandy, Caen 14000, France; # Division of Oncology, Department of Medicine and Department of Pathology, 6429 Stanford University School of Medicine, Stanford California 94305, United States

**Keywords:** nanomedicine, neuroprotection, blood–brain barrier, glial cells, thrombolytics

## Abstract

Recombinant tissue-type plasminogen activator (tPA) is the only approved thrombolytic drug for acute ischemic stroke, a condition associated with severe disabilities and high mortality. However, when the blood–brain barrier (BBB) is damaged, tPA can exacerbate cerebral injury and increase the risk of hemorrhagic transformation, limiting its use to a small subset of patients. To address this challenge and minimize extravascular accumulation, we combined tPA with micrometer-sized particles (DPN). We then tested their safety and neuroprotective effects. After a 1 h transient occlusion of the middle cerebral artery, free tPA, tPA-DPN, or saline was administered to assess mice survival, neurological behavior, and infarcted area extent. Free-tPA exacerbated brain damage, resulting in a modest 10% survival rate at 24 h post intervention. Conversely, tPA-DPN displayed a far better prognosis, with a 75% survival rate comparable to that of saline. No statistical differences were documented between tPA-DPN and saline for the Activity Score and the Neurological Severity Score. tPA-DPN did not increase lesion volume or BBB permeability, unlike free-tPA, which led to an over 2-fold enlarged lesion volume and 50% higher BBB permeability. The safety profile of tPA-DPN is attributed to the robust conjugation of tPA onto DPN and the lack of DPN extravasation, resulting in negligible cerebrovascular damage of free tPA and glial and neuron impairment. The vascular confinement of tPA linked to microscopic particles reduces drug side effects and represents a valuable strategy for safe and effective tPA delivery, even in the postacute stroke phase.

## Graphical abstract

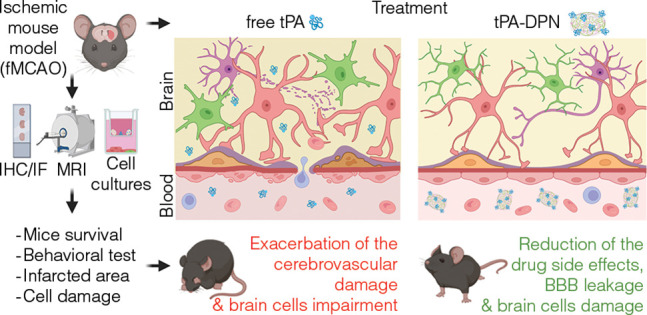

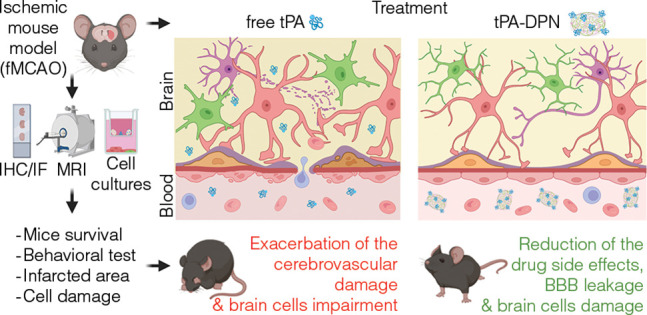

## Introduction

Stroke is an acute episode of focal dysfunction of the brain, retina, or spinal cord, associated with local infarction or hemorrhage.^1^ With over 12 million new cases annually, stroke is the most fatal cerebrovascular disease worldwide and the second leading cause of death globally, accounting for around 11% of deaths (6.5 million) every year.^2^ Patients surviving a stroke often suffer moderate-to-severe neurological deficits, with a significant fraction of patients losing their independence in performing daily activities. Currently, in acute ischemic stroke, three therapeutic options are available: intravenous administration of Alteplase, a recombinant tissue-type plasminogen activator (tPA); thrombectomy, an endovascular procedure aiming at removing blood clots by mechanical retrieving or aspiration; and a combination of both.^3^ The efficacy of these approaches is time-dependent, with better outcomes when tPA is given within 3 h from stroke onset.^3^ Clinical practice encourages the use of Alteplase within a 4.5 h window^4^ and requires expensive imaging procedures for later administrations (4.5 to 9.0 h), with a risk/benefit ratio that increases rapidly after 6 h from the event.^5^ Within the first 6 h, thrombectomy can be performed, with guidelines recommending the combination with Alteplase only in patients at low risk of bleeding.^6^ With all these limitations, the current portfolio of clinically approved stroke therapies benefits less than 20% of the patients.^7^ Only a few clinical trials are evaluating the efficacy of new thrombolytics, such as Tenecteplase, a modified tPA that appears to preserve the efficacy of Alteplase but with improved pharmacological parameters, or other agents, including nanomedicines.^8–10^

Nanotechnologies offer a wide range of solutions to overcome the limitations of current therapies.^11–13^ Furthermore, nanotechnologies can combine multiple active agents (molecules for targeting and drugs) and finely modulate their release. Despite the modest clinical trial activity on nanothrombolytics, several nanomedicines such as liposomes, inorganic nanoparticles, polymeric nanoparticles, and microbubbles have demonstrated benefits in preclinical studies.^9,14^ For example, loading thrombolytic drugs inside the aqueous core of liposomes increased both their half-life and their efficacy. An evolution of this system is echogenic liposomes, which combine drug loading with ultrasound-sensitivity for controlled release and mechanical destruction of blood clots.^13^ Inorganic nanoparticles are interesting for imaging and theranostic approaches. For example, tPA-loaded iron oxide nanocubes combined a clot-targeting contrast agent for MRI with thermal sensitivity and pharmacological activity. Both in vitro and in vivo studies have shown that tPA-nanocubes exhibit higher clot dissolution rates compared to free-tPA alone, with synergistic effect observed when hyperthermia is present.^15^

Polymeric nanoparticles are a different class of particles with a size between 10 and 1000 nm that can be synthesized with different shapes and physicochemical properties. Among several examples, a notable recent development is provided by the polymeric particles developed by Chauvierre and collaborators, who designed and preclinically tested ultrasound-responsive, stable, biocompatible microbubbles made of polyisobutyl cyanoacrylate copolymerized with fucoidan, a molecule known to efficiently target activated endothelium. Their study found that tPA-loaded fucoidan microbubbles were 50% more efficient than free tPA and required only one-tenth of the standard dose to resolve the stroke.^14^ Moreover, Colasuonno et al. suggested the use of microscopic particles—discoidal polymeric nanoconstructs (DPN)—as vehicles for Alteplase.^16^ DPN are made of poly­(lactic-*co*-glycolic acid) (PLGA) and polyethylene glycol (PEG), both biocompatible and clinically approved polymers. DPN were successfully tested for vascular delivery of imaging and therapeutic molecules against tumors.^17^ The authors documented in previous studies that tPA-DPN outperform free-tPA, showing higher blood clot dissolution rates.^16^

Recent clinical research efforts aim at extending the therapeutic window for tPA administration while minimizing its adverse side effects.^18^ The primary issues associated with tPA administration arise from its extravascular cerebral effects, which significantly elevate the risk of hemorrhagic transformation and mortality.^3,6,7^ In this work, we propose that the safety of tPA administration can be improved by delivering it via vascular-confined micrometric particles. As a proof of concept, we chose the tPA-DPN, which were recently developed in our lab for various applications.^17^ tPA-DPN are characterized thoroughly both in vitro (physical–chemical properties and thrombolytic potential) and in vivo in a severe occlusion/reperfusion middle cerebral artery (MCAO) mouse model. Behavioral and survival analyses on mice show the positive effect of tPA conjugation to DPN compared with that of the drug alone. Histological studies on lesion size and immunoglobulin G (IgG) extravasation, corroborated with MRI, correlate the positive effects observed in behavioral and survival experiments with a reduced infarcted area and BBB leakage. In vitro experiments on primary cultures of murine microglial and astrocytic cells highlighted the different susceptibilities of the two cell types to tPA.

## Results

### Synthesis and Characterization of tPA-DPN

DPN were synthesized by a top–down fabrication approach, originally described by Key and collaborators,^19^ resulting in the formation of 1000 nm × 400 nm (diameter × height) discs made of intercalating PLGA and PEG-DA chains (Figure 1A). The clinically approved recombinant tissue-type plasminogen activator Alteplase was covalently bound to the DPN surface upon activation of the carboxylic acid termini of the PLGA chains using the EDC/NHS reaction (Figure 1B).^16^ The reaction binds on the carboxylic groups of the PLGA an amine-reactive NHS ester leading to a stable bond in the presence of a primary amine, like for the N-terminal of tPA. Scanning electron microscopy (SEM) confirmed the homogeneity of each batch (Figure 1C) and documented the characteristic discoidal shape of the particles. DPN size distribution and surface zeta (ζ)-potential were evaluated via the Multisizer 4E Coulter Particle Counter and Zetasizer Nano Instrument, respectively. The modest difference in modal values between the size of the DPN alone (754 nm) and that of tPA-DPN (681 nm) confirmed that the addition of the thrombolytic protein has a negligible effect on the particle geometry, as also documented by the distribution profile returned by the Multisizer Coulter counter (black vs orange curves, Figure 1D). Conversely, the addition of tPA was associated with a marked increase in surface electrostatic charge from −23.31 ± 3.37 mV for DPN alone to 19.62 ± 4.17 mV for tPA-DPN (*p* < 0.0001), as assessed via the Zetasizer Nano Instrument (Figure 1E). This should be ascribed to the activation of the carboxylic acid groups on the DPN surface via the EDC/NHS reaction and partial neutralization with their conjugation to the N-terminus of tPA. The efficiency of the conjugation of tPA to the DPN surface was evaluated via the BCA assay. The total protein amount quantified on tPA-DPN was compared to the original tPA input, returning a bioconjugation efficiency (BE) of 63.66 ± 8.16% (Figure 1F). The term “Bioconjugation Efficiency (BE)” is analogous to the more conventional “Encapsulation Efficiency (EE)”. BE is calculated as the ratio of the amount of tPA conjugated to the DPN after washing and purification (final amount of tPA, determined via BCA assays) to the initial amount of tPA incubated with the DPN. This measure reflects the conjugation efficiency or, in other words, the effectiveness of associating tPA with DPN. As expected, no tPA was detected by the BCA assays on DPN-alone (unpublished data). Additionally, we correlated the particle concentration with the protein amount to use comparable doses of DPN, free-tPA, and tPA-DPN in each experiment. We obtained the value of 4.85 ± 1.56 million tPA-DPN per microgram of tPA (Figure 1G). It is here important to highlight that, at the end of each DPN batch synthesis, we performed the above physicochemical and biological characterizations to assess the quality of the nanoconstructs before conducting any experiment.

**Figure 1:**
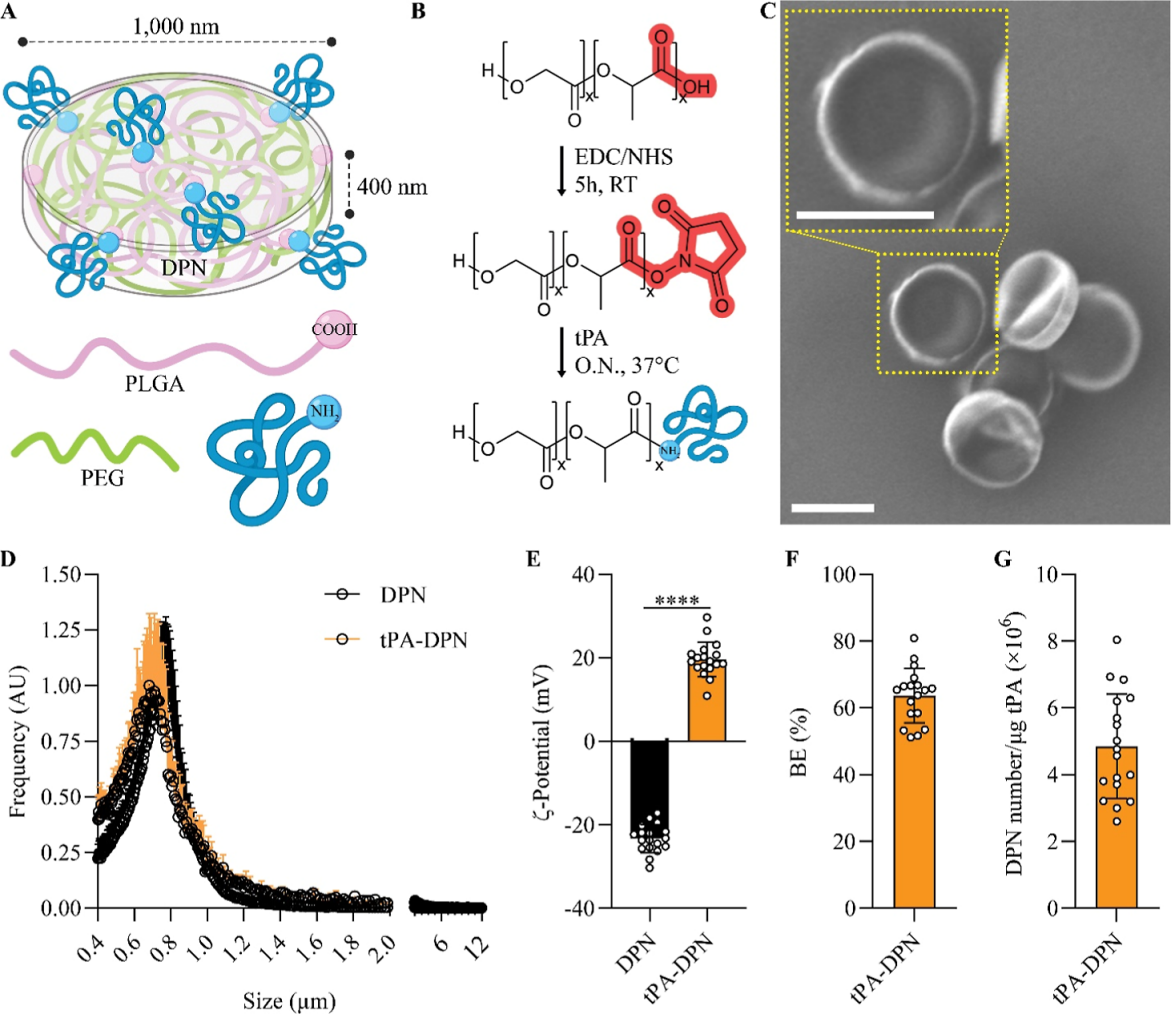
Characterization of DPN loaded with tissue-type plasminogen activator (tPA). (A) Schematic representation of DPN loaded with tPA. (B) Synthetic route for the tPA conjugation on PLGA molecules. (C) Representative images of the morphology of tPA-DPN after tPA-conjugation obtained by scanning electronic microscopy (scale bar 1 μm). (C) is 6000× magnification acquisition. The inset shows high magnification detail with a single particle (scale bar 1 μm). (D) Multisizer size distribution and (E) DLS ζ-potential measure of DPN and tPA-DPN (*n* = 18, ****, *p* < 0.0001, two-tailed unpaired *t*-test). (F) tPA bioconjugation efficiency (BE), expressed as the percentage of the tPA amount measured on tPA-DPN compared with the tPA input. Concentration amounts were evaluated through bicinchoninic acid assay (BCA assay) (*n* = 18). (G) Quantification of the tPA amount per millions of DPN by the BCA assay (*n* = 18).

### In Vitro Thrombolytic Activity of tPA-DPN

tPA-DPN were further assessed in vitro by the thrombolytic “halo’” assay, adapted from a previous work.^20^ (Figure 2A and B). tPA-DPN displayed a thrombolytic behavior comparable to that of free-tPA, while empty DPN showed no activity. This is highlighted in Figure 2C that shows the dissolution profiles from the halo clot assay as a function of the time of incubation: the free-tPA curve (blue circles) and tPA-DPN curve (orange circles) overlapped for the entire duration of the experiments (120 min), whereas the DPN alone without any tPA (black circles) did not present any lytic activity. More quantitative information can be derived by considering three relevant parameters, as defined in Figure 2B, namely, the maximum clot lysis rate (CLR_max_), which corresponds to the maximum positive slope value of the degradation profile; the activation time (*A*_t_), which represents the time (in minutes) when the slope of the degradation profile exceeds 1; and *T*_0.5_, which corresponds to the time (in minutes) needed to reach 50% lysis. A direct comparison between free-tPA and tPA-DPN for all three listed parameters is provided in Figure 2D–F, respectively. No statistically significant differences were observed for all three parameters. CLR_max_, *A*_t_, and *T*_0.5_ were all zero for the empty DPN, confirming again the lack of any thrombolytic activity for the microparticles alone. In addition, *T*_CLRmax_ was registered to be higher for tPA-DPN compared to free tPA (16.00 ± 5.02 vs 26.00 ± 9.78, with *p* = 0.05, Supporting Figure S2).

**Figure 2:**
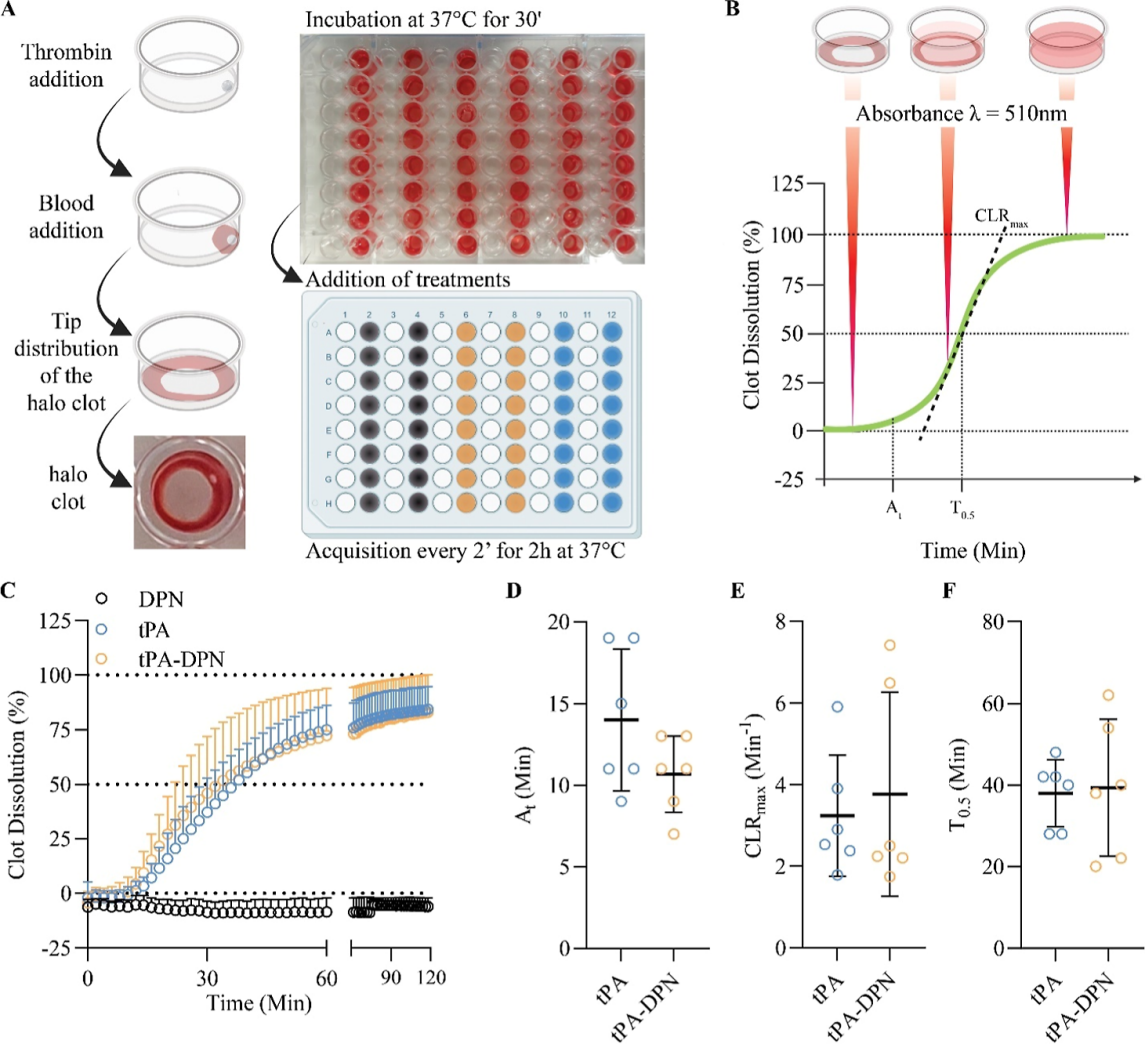
In vitro thrombolytic efficacy of tPA-DPN by halo assay. (A) Schematic representation of the “halo” thrombolytic test. (B) Longitudinal real-time acquisition allows the calculation of additional parameters “activation time” (*A*_t_), CL*R*_max_, and *T*_0.5._. (C) In vitro thrombolytic plot with free-tPA, DPN, and tPA-DPN clot dissolution profile. (D–F) show the additional parameters evaluated within the test (*n* = 6, two-tailed unpaired *t*-test).

Lastly, the reactive oxygen species (ROS) production over thrombolysis has been evaluated through “halo” assay, using the DCFH_2_-DA probe. No ROS production was detected over thrombolysis by tPA-DPN (*p* = 0.9994) or DPN alone (*p* = 0.9822) (Supporting Figure S12).

### Survival and Neurological Scores in fMCAO Mice Treated with tPA-DPN

To evaluate the safety profile of tPA-DPN over free-tPA, we used a mouse model resulting from the transient occlusion and reperfusion of the middle cerebral artery (MCA) via the insertion of a filament—the fMCAO model.^21^ Specifically, a silicon filament is introduced through the carotid artery to reach and block the blood flow at the origin of the MCA (Figure 3A). The extent of cerebral damage is related to the duration of the occlusion. In this work, the filament was retracted only after 1 h of occlusion, thus inducing pronounced tissue damage in the cortical and striatal areas. This severe preclinical stroke model allows us to document more effectively the side effects associated with the use of free-tPA.^22,23^ Treatments were administered 20 min post filament removal and included free-tPA (10 mg kg^–1^) or tPA-DPN (10 mg kg^–1^ tPA equivalent dose) with a 10% bolus followed by a 90% slow infusion for over 20 min (Figure 3A), simulating the clinical administration routine.^3,6^ After 24 h, all the mice that survived the procedures were subjected to neurological assessment via the activity scoring (AS), ranging between 0 and 1, with 1 identifying healthy mice; and the neurological severity scoring (NSS), ranging between 0 and 6, with 0 identifying healthy mice. First, we observed a striking difference in survival among the three treatment groups (Figure 3B, Supporting Figure S1A). Almost 90% of the mice treated with free-tPA died before 24 h, returning a survival rate of only 12.5%. This result clearly confirms the severity of the preclinical stroke model considered in the present work. On the contrary, mice treated with tPA-DPN and saline exhibited a survival rate of 71.4% and 87.5%, respectively (*p* = 0.7917—saline vs tPA–DPN; *p* = 0.0069—saline vs tPA; *p* = 0.0299—tPA vs tPA-DPN). Similarly, Kaplan–Meier curves (Supporting Figure S1A) highlighted the statistical differences in average survival among all the groups (*p* = 0.0019, Log-rank Mantel–Cox test): saline vs tPA (*p* = 0.0016, Log-rank Mantel–Cox test) and tPA vs tPA-DPN (*p* = 0.0143, Log-rank Mantel–Cox test) but no differences between saline vs tPA-DPN (*p* = 0.3995, Log-rank Mantel–Cox test). The sham mice that underwent surgical intervention without occlusion (as the filament was immediately removed after reaching the MCA), had 100% survival (Supporting Figure S1B). Second, the neurological tests confirmed the reduction of toxicity associated with the administration of tPA-DPN in the presence of a stroke as opposed to free-tPA. Specifically, for the AS (Figure 3C), the tPA-DPN returned a value comparable to that of the saline group (*p* = 0.5863, ns), whereas the tPA group was associated with a much lower score given the poor animal survival (*p* = 0.0079 vs free-tPA vs saline; *p* = 0.0544 vs free-tPA vs tPA-DPN). A similar trend was observed for NSS (Figure 3D). The tPA-DPN outperformed the free-tPA group (*p* = 0.0394, ns −free-tPA vs tPA-DPN) returning a score comparable to that of the saline group (*p* = 0.4283 −saline vs tPA-DPN). In the sham animals, survival rate and neurological testing were not affected by any of the treatments, returning survival rates of 100%, activity average scores close to 1 and neurological severity scores close to 0 (Supporting Figure S1B–D). Note that empty circles were used to identify animals that survived the procedure, whereas filled circles were associated with animals that died within 24 h post procedure.

**Figure 3:**
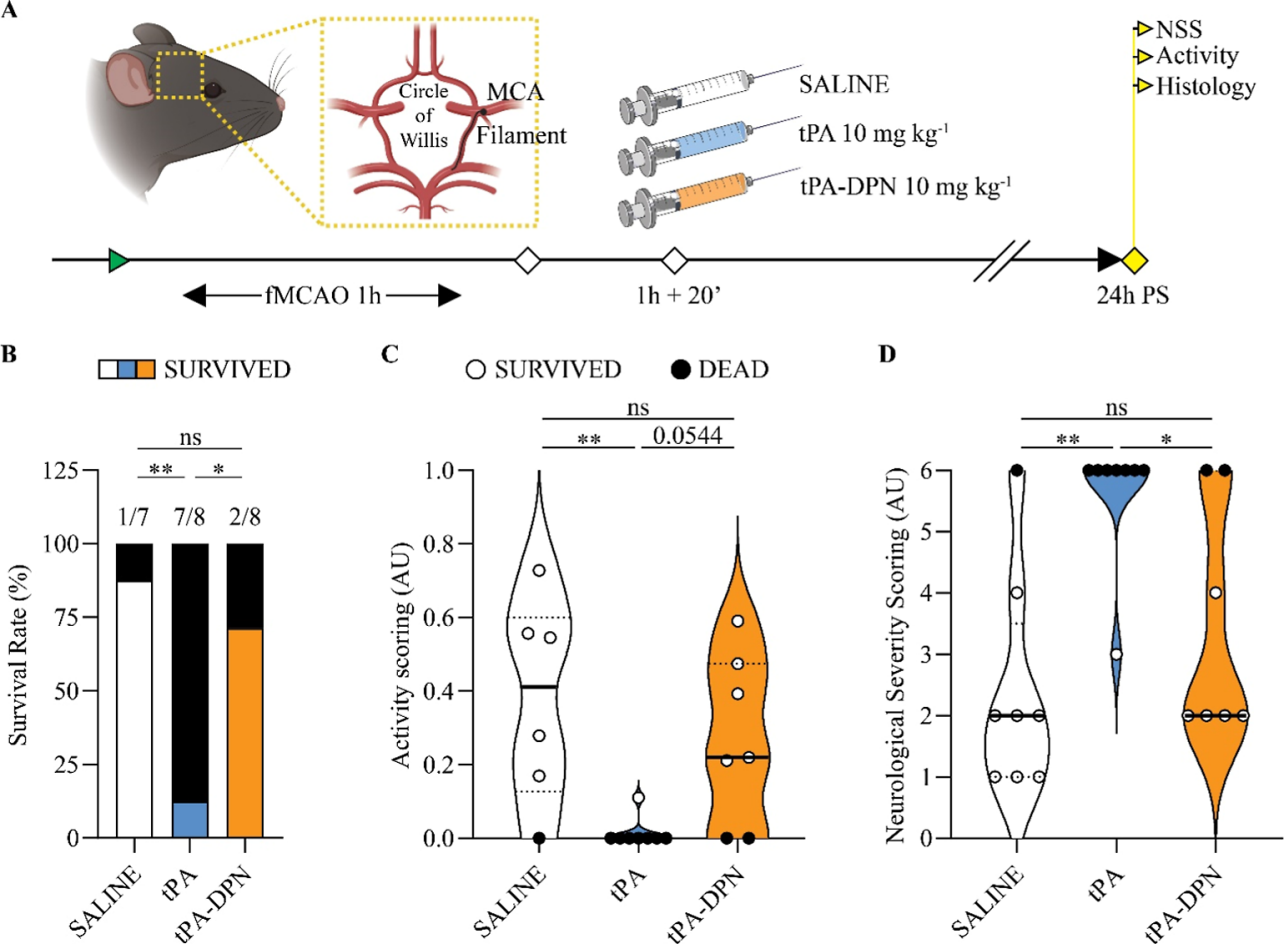
tPA-DPN neuroprotective evaluation in the preclinical model of transient Middle Cerebral Artery (MCA) occlusion by filament. (A) Schematic of the animal model and description of the experimental end points. Briefly, 24 h before the surgery all animals were tested for behavioral scoring to check the initial status of the mice. Activity and Neurological Severity scoring were repeated 24 h post-occlusion (PS; fMCAO1h) and treatment (saline, tPA 10 mg kg^–1^ or tPA-DPN 10 mg kg^–1^). (B) Survival rate 24 h PS, after treatment with just vehicle (saline), free-tPA 10 mg kg^–1^, and tPA-DPN 10 mg kg^–1^. (C) Activity scoring and (D) neurological severity scoring (NSS). Black symbols refer to animals found dead after 24 h. Results are expressed as mean ± SD (*n* ≥ 6; *p < 0.05, **p < 0.01, respectively; one-way ANOVA, with Tukey correction).

### Size of the Lesion and Histological Analyses in fMCAO Mice Treated with tPA-DPN

Following the behavioral tests, mice were sacrificed, and their brains were isolated, photographed, and prepared for a series of histological analyses. The Supporting Figure S3 presents representative images of mouse brains taken right after sacrifice. Interestingly, vascular damage and tissue darkening can be readily observed in the left hemispheres, especially for those mice treated with free tPA. Then, for the first histological analysis, the harvested brains were prepared for Cresyl Violet (CV) staining (a schematic of the histological sample preparation and quantification is shown in Supporting Figure S4 with a detailed description in Methods—Imaging Analysis). In CV staining, unstained whitish areas are associated with the infarcted tissue (left side of the brain, Figure 4A). From these sections, the volume of the lesion V_TOT_^lesOE^ was quantified for all three experimental groups, returning values for the free-tPA group (99.98 ± 23.13 mm^3^) much higher than those for the saline (54.39 ± 15.85 mm^3^) and tPA-DPN (60.30 ± 14.99 mm^3^) groups (Figure 4B). Notably, while a statistically significant difference was documented between free-tPA and saline (*p* = 0.0038) as well as free-tPA and tPA-DPN (*p* = 0.010), no difference was observed between the saline and tPA-DPN (*p* = 0.8305, ns), suggesting once again the improved safety profile of the tPA-DPN over free-tPA. It is also important to observe that no difference in overall brain volume (Supporting Figure S5A) and edema (ipsilateral/contralateral hemispheres ratio) (Supporting Figure S5B) was documented among the treatment groups in the presence of stroke. Conversely, as expected, an increase in edema was observed when comparing the fMCAO with the sham groups (no stroke) (Supporting Figure S5B). Furthermore, the volume of the lesion was plotted along the distance from the Bregma, as shown in Figure 4C and detailed in Figure 4D. This data representation allows one to more accurately assess the distribution of the infarcted volume throughout the brain and aims to emphasize the potential impact on various cerebral regions. From these plots, the area under the curve (AUC) was quantified as a way to measure the overall volume of the lesion (Figure 4E) for the three different experimental conditions. The data confirmed a larger infarcted area for the free-tPA group. Specifically, statistically significant differences were computed for free-tPA vs saline (*p* = 0.0125) as well as free-tPA vs tPA-DPN (*p* = 0.0282), whereas no difference was observed for the saline vs tPA-DPN comparison (*p* = 0.9113, ns).

**Figure 4:**
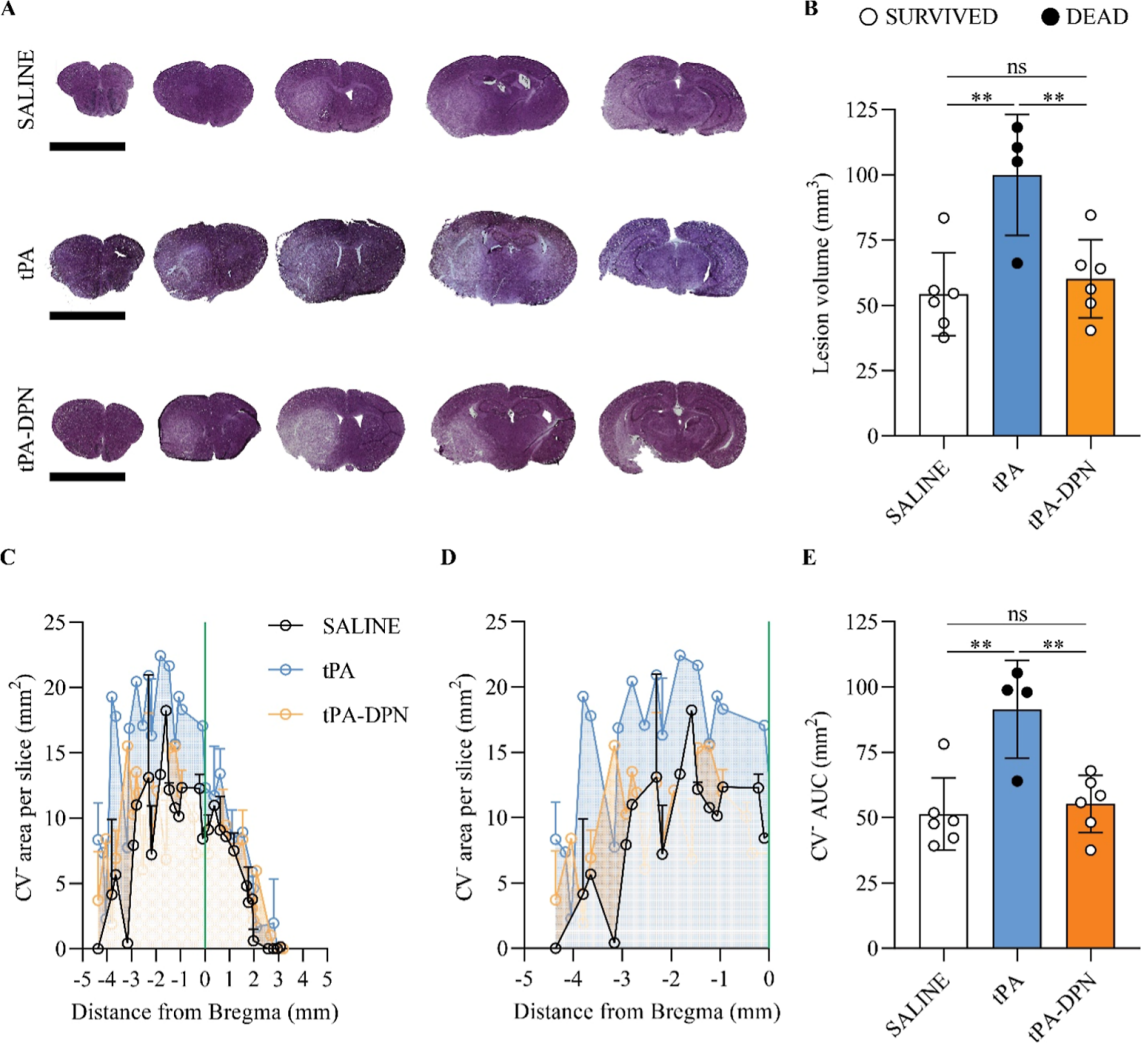
Histological analysis for lesion size. (A) Representative images from the three different experimental groups, stained with Cresyl Violet, to highlight the lesion area (white), against the undamaged areas (violet). The scalebar, in the lower left of each panel, is 5 mm. (B) Lesion volume, expressed in mm^3^, was obtained measuring the unstained Cresyl Violet negative brain area over the sections. Black symbols refer to animals found dead. Results are expressed as mean ± SD (*n* > 4; **p < 0.01; one-way ANOVA, with Tukey correction). (C) Pattern of the ischemic lesion area over the brain and among the different conditions. Each section was localized inside the brain and over the antero-posterior axes, using the Bregma point as center (0). (D) Detail of the posterior part of the brain (from −5 mm to 0 mm from the Bregma point) for the CV^–^ area distribution. (E) Area Under the Curve (AUC) calculations were performed individually for each sample. AUC measurements were plotted for each experimental group as mean ± SD (*n* = 6; **p < 0.01, one-way ANOVA, with Tukey correction).

The size of the lesion was also assessed longitudinally at 3, 6, and 24 h post stroke via MR imaging, using a specific *T*_2_-weighted acquisition (Supporting Figure S6). Unfortunately, this analysis was inconclusive as the free-tPA treated mice did not survive up to 24 h post procedure, preventing any meaningful statistical analysis. Nonetheless, the MRI data document once again the increased safety of the tPA-DPN over free-tPA and the severity of the model that induced lesion volumes larger than 50 mm^3^ already within the first 3 h post occlusion. It is here just important to note that other preclinical stroke models have typically lesion volumes in the order of 20 to 30 mm^3^.^8^

### Blood–Brain Barrier Permeability in fMCAO Mice Treated with tPA-DPN

The status of the BBB was characterized histologically by quantifying the extravascular accumulation of native IgG, whose presence in the brain parenchyma is minimal if not negligible under normal conditions (intact BBB), and via MR imaging following the systemic administration of a gadolinium-based contrast agent. First, representative images after IgG staining showed the presence of a wide green-positive area (left side of the brain) in all three treatment groups (Figure 5A). A much higher IgG accumulation in the brain parenchyma was detected after free-tPA administration (69.84 ± 12.19 mm^3^) in comparison to that with saline (49.51 ± 14.36 mm^3^) or tPA-DPN (50.20 ± 14.53 mm^3^) (Figure 5B). Once again, while a statistically significant difference was documented between free-tPA and saline (*p* = 0.0323) as well as free-tPA and tPA-DPN (*p* = 0.0390), no difference was observed between saline and tPA-DPN (*p* = 0.9952, ns), confirming the augmented safety profile for tPA-DPN over free-tPA. Even in these studies, all tested animals presented no difference either in the total brain volume or edema (Supporting Figure S5D,E). However, a comparison between the fMCAO groups and the sham animals, as expected, documented an increase in edema (Supporting Figure S5E). Moreover, by analyzing the stained sections, density plots were generated for the IgG extravascular accumulation along the distance from the Bregma (Figure 5C,D). This data documented a significant increase in IgG extravascular accumulation for free-tPA over tPA-DPN (*p* = 0.0260), while no significant difference was observed between tPA-DPN and saline (*p* = 0.8120, ns) (Figure 5E). In this case, no significant difference was documented between saline and free-tPA too (*p* = 0.1044).

**Figure 5:**
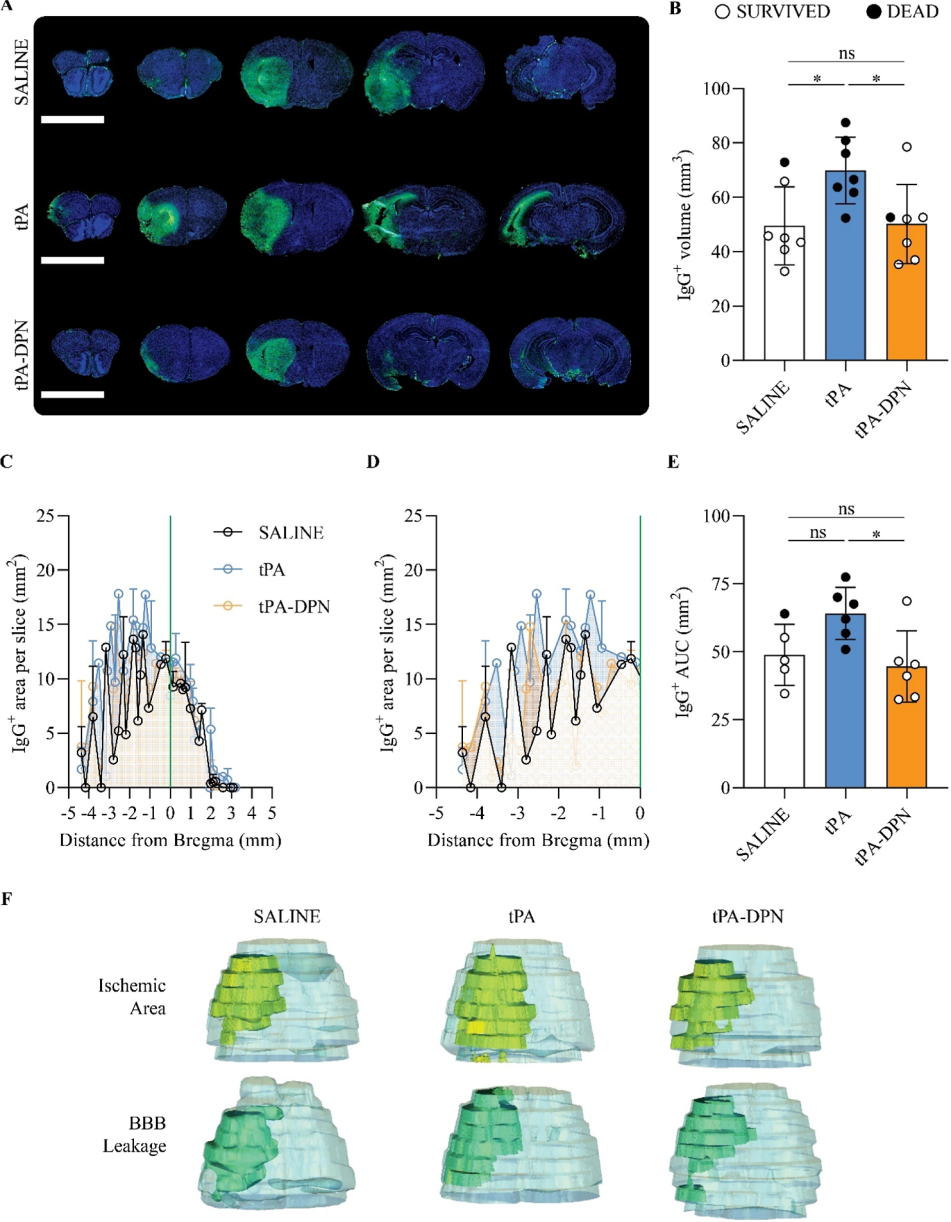
Histological analysis for blood–brain barrier leakage. (A) Representative IgG-stained images, displaying BBB leakage. IgG presence is visible as green signal, while blue is the counterstaining of the cell nuclei. The scalebar, in the lower left of each panel, is 5 mm. (B) IgG^+^ volume, expressed in mm^3^, was obtained measuring the brain area over the sections positive to the fluorescent IgG signal (*n* = 6; *p < 0.05; one-way ANOVA, with Tukey correction). (C) Pattern of the IgG-positive area over the brain and among the different conditions. Each section was localized inside the brain and over the antero-posterior axes, using the Bregma point as center (0). (D) Detail of the posterior part of the brain (from −5 mm to 0 mm from Bregma point) for the IgG area distribution. (E) area under the curve (AUC) calculations for the IgG^+^ profiles, performed individually for each sample. AUC calculation expressed as mean ± SD (*n* ≥ 5; *p < 0.05, one-way ANOVA, with Tukey correction). (F) 3D image reconstruction from histological samples, with volume overlapping among whole brain (light blue), lesion size (yellow), or BBB leakage (green).

To demonstrate the spatial colocalization between the lesion and the area with enhanced vascular permeability (IgG extravasation), a 3D volume reconstruction was performed from the histological sections using an ad hoc algorithm (Supporting Figures S7 and S8). The 3D reconstructions confirmed a good overlap between the damaged tissue (Ischemic Area) and the vascular hyperpermeable zones (BBB Leakage) for all three treatment groups (Figure 5F–Supporting Table S2).

To further verify the impact of the different treatments on vascular permeability after filament removal, we used a clinically relevant imaging modality—Magnetic Resonance Imaging (MRI).^24^ Vascular permeability experiments by MR imaging were performed at 3 h poststroke, following the systemic administration of a gadolinium-based contrast agent (Figure 6A). *T*_1_-weighted sequences revealed the signal associated with the extravasation of the contrast agent as bright hyperdense areas (Figure 6B). A clearly visible signal from the infarcted area was detected for the free-tPA group with a 10.20 ± 1.87 mm^3^ volume, while saline and tPA-DPN returned much lower values corresponding to 3.622 ± 0.9157 mm^3^ and 0.5443 ± 0.9427 mm^3^, respectively. A statistically significant difference was documented between free-tPA and saline (*p* = 0.0016) as well as between free-tPA and tPA-DPN (*p* = 0.0002) (Figure 6C), while no difference was detected between saline and tPA-DPN (*p* = 0.06550, ns). The corresponding 3D volume reconstruction of the MRI data is shown in Figure 6D, for all three experimental groups.

**Figure 6:**
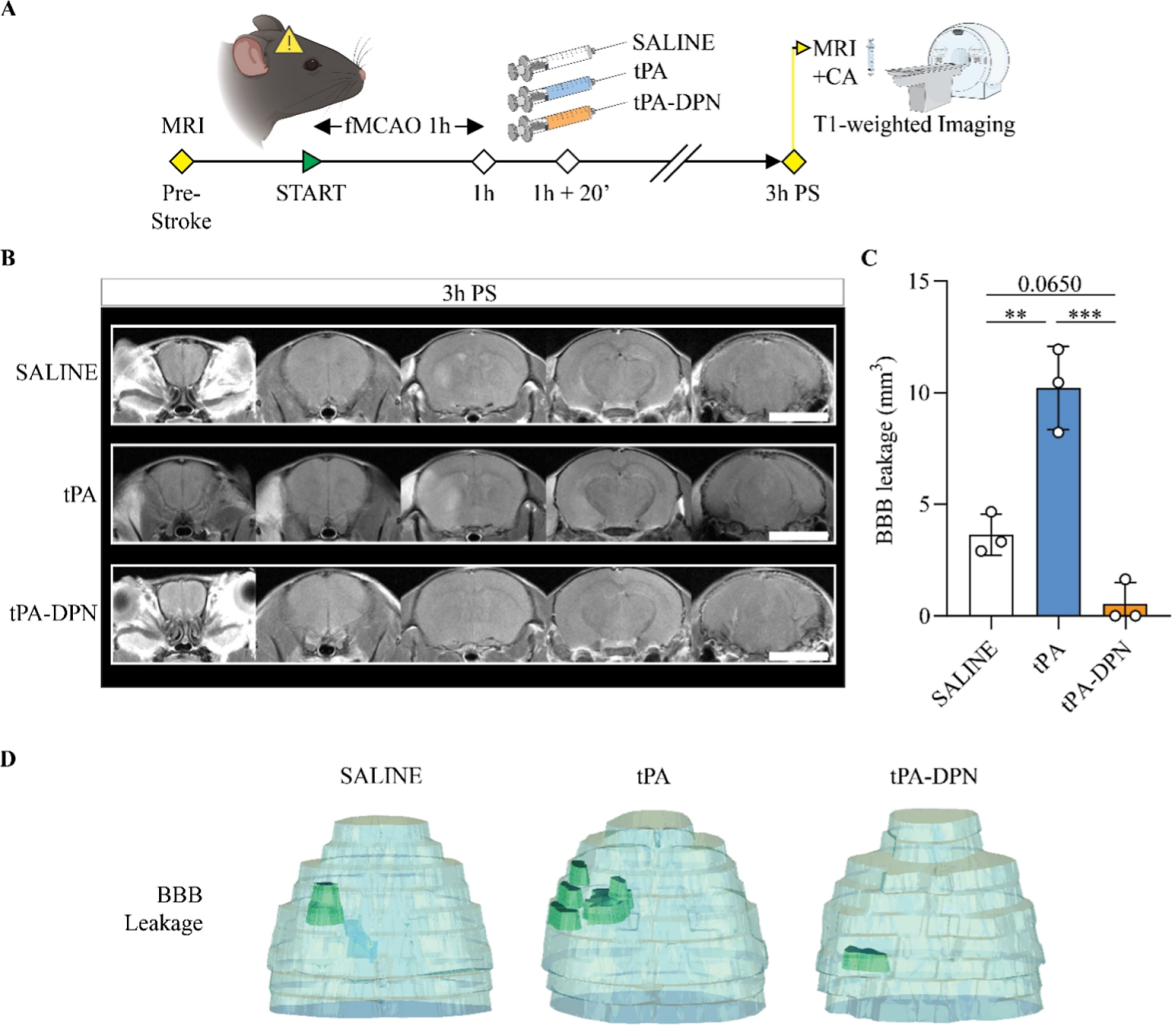
T1-weighted MRI analysis for blood–brain barrier leakage. (A) Schematic representation of the experimental plan for BBB leakage MRI investigation. 24 h before the surgery, T1-weighted (T1w) imaging was performed to check the initial status of the mice. The contrast agent (ProHance) was systemically injected 15 min before the imaging. MRI T1w imaging with contrast agent was repeated 3 h post-occlusion (PS; fMCAO 1h) and treatment (saline, tPA 10 mg kg^–1^ or tPA-DPN 10 mg kg^–1^). (B) Representative T1-weighted images after the injection of the contrast agent (CA, ProHance) 3 h PS. Scalebar 5 mm. (C) Comparison of the BBB leakage among experimental groups. Results are expressed as mean ± SD (*n* = 3; **p < 0.01, ***p < 0.001, respectively; one-way ANOVA, with Tukey correction). (D) 3D image reconstruction from MRI acquisitions, with volume overlapping among whole brain (light blue) and BBB leakage (green).

### Effect of tPA-DPN on Microglial Cells, Astrocytes, and Neurons

It is well accepted that free-tPA can reach the cerebral parenchyma by crossing the damaged BBB and, thus, affect different brain cells—mostly microglia and astrocytes—that are sitting next to the vascular compartment.^25–28^ To assess the effect of free-tPA on individual cell populations, we established primary mouse cell culture of microglial cells, astrocytes, their co-culture, and neurons. First, different concentrations of free-tPA, ranging from 1 μg mL^–1^ (14.3 nM) to 400 μg mL^–1^ (5.7 μM), were used to determine cell metabolism and estimate the corresponding IC_50_ values. At 24 h post treatment, astrocytes were observed to be more susceptible than microglial cells to free-tPA exposure (Figure 7A and B). Specifically, we determined an IC_50_ value of 121.1 μg mL^–1^ (1.7 μM) for microglial cells and 18.3 μg mL^–1^ (261.6 nM) for astrocytes. Interestingly, the toxicity of free-tPA was partially mitigated when cells were coseeded, with about 30% microglial cells and 70% astrocytes (glia cells), returning an IC_50_ value of 92.8 μg mL^–1^ (1.3 μM) (Figure 7C). Neurons showed a slightly higher susceptibility to tPA treatment than the mixed glial cells population, as revealed by IC_50_ assays (Supporting Figure 11A; neurons IC_50_ of 77.26 μg mL^–1^ vs mixed glial cells IC_50_ of 92.8 μg mL^–1^). Additionally, neurons seemed more resistant than astrocytes and less resistant than microglial cells.

**Figure 7:**
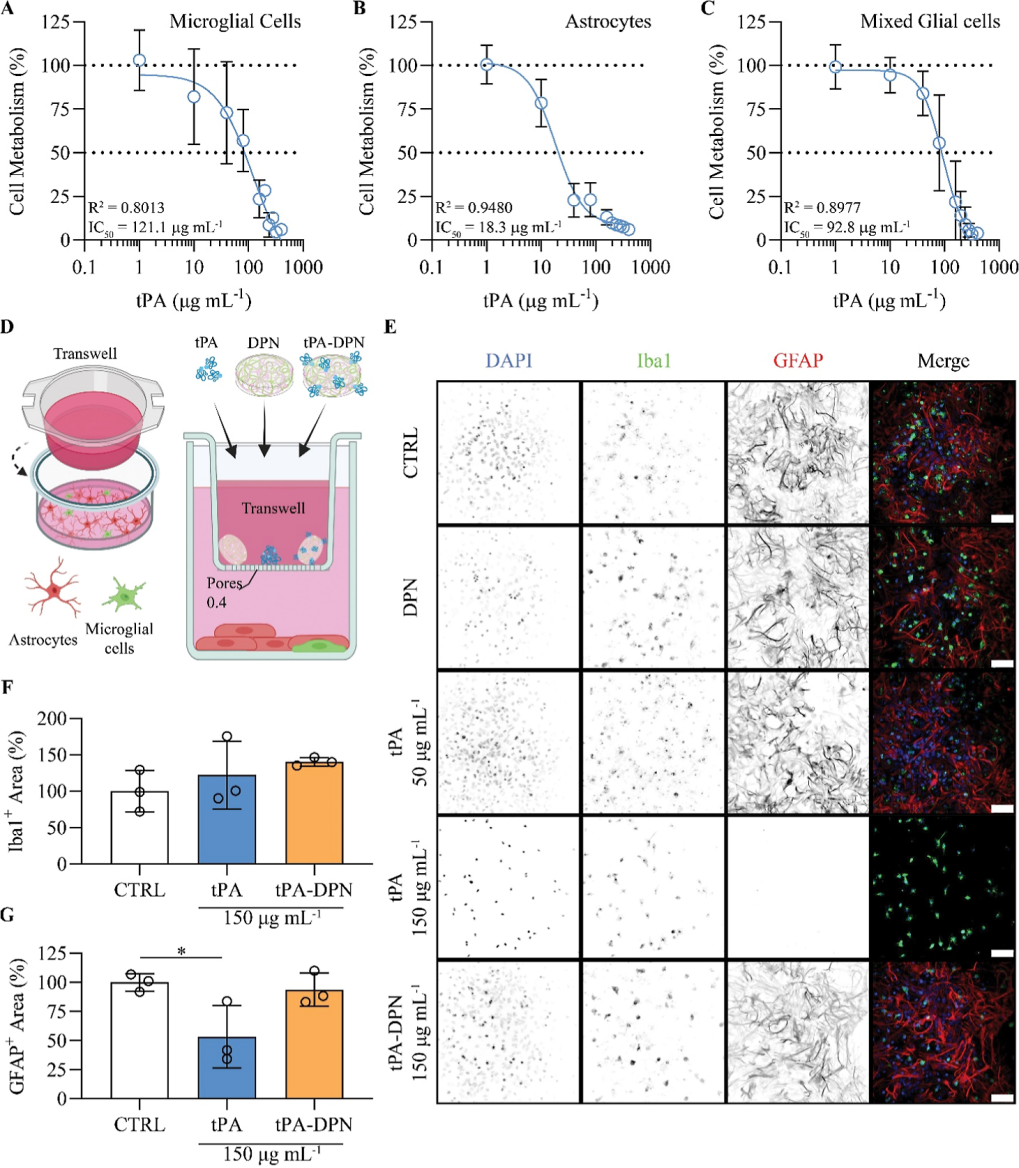
In vitro effect of free-tPA and tPA-DPN on primary glial cells. (A), (B), and (C) show the impact on cell metabolisms of 24 h free-tPA treatment at different concentrations. (D) Schematic representation of the in vitro model composed by a mixed co-culture of glial cells in the presence of a porous barrier (Transwell chamber, with 0.4 μm pore-size). The different treatments (free-tPA 50 and 150 μg mL^–1^, tPA-DPN 150 μg mL^–1^, DPN or complete media) were loaded inside the apical part of the inset. (E) Representative confocal images showing cells morphology change after the different treatments. Here, nuclei were stained with DAPI (blue), while Iba1 and GFAP were in green and red, respectively. Black and white images of each row of the panel show the single marker expression for each area acquired, with the last color image displaying the composite image with all the markers. Images were acquired at 20× magnification (scale bar: 100 nm). (F,G) Immunofluorescence analysis on Iba1 and GFAP positive area inside the region of interest, expressed as percentage. Results are expressed as mean ± SD (*n* = 3; *p < 0.05; one-way ANOVA, with Tukey Correction).

Starting from these results, the potential impact of low and high concentrations of free-tPA and tPA-DPN was evaluated on glial cells (low concentrations: 50 μg mL^–1^ or 0.7 μM; high concentration: 150 μg mL^–1^ or 2.1 μM). Glial cells (*i.e.,* astrocytes and microglia) are key regulators of BBB permeability and the first cells exposed to blood-borne agents. To perform these tests, we adopted a transwell-based setup to simulate a compromised blood–brain barrier with large pores documenting vascular hyperpermeability—hyperpermeable BBB transwell model. Treatments were administered within a transwell system comprising a 400 nm porous membrane separating the basolateral chamber, with the glia cells, from the apical chamber loaded with free-tPA, DPN alone, and tPA-DPN (Figure 7D). Note that DPN alone and tPA-DPN could not diffuse in the bottom chamber through the membrane given their characteristic morphology −1000 nm × 400 nm discs. At 24 h post treatment, the glial cells were stained for Iba1 (Ionized calcium Binding Adaptor Molecule 1, as a microglia marker) and GFAP (Glial Fibrillary Acidic Protein, as an astrocytes marker) and with DAPI to identify the cell nuclei. Figures 7E and Supporting Figure S9 show the results of the fluorescent immunostaining for the different groups and treatment conditions. At low tPA concentrations (50 μg mL^–1^ or 0.7 μM), no significant differences were observed in terms of the Iba1 and GFAP expressions. However, at sufficiently high tPA concentrations (150 μg mL^–1^ or 2.1 μM), a dramatic decrease in the GFAP marker was documented for the free-tPA-only group. The quantification of the Iba1 and GFAP markers for the high tPA concentration confirmed the above observations, returning a modest and statistically not relevant variation in Iba1 expressions (Figure 7F) as well as the significant impact on astrocytes (Figure 7G) (DPN alone vs free-tPA, *p* = 0.0435). Notice that no effect of free-tPA on glial cells was documented at lower concentrations of 50 μg mL^–1^, or 714.3 nM (Figure 7E). In a similar transwell setup, a dose of tPA-DPN (150 μg mL^–1^), two times higher than the tPA IC_50_ for the neurons, did not affect cell viability, whereas high doses of free tPA (150 μg mL^–1^) induced toxic effects, leading to a dramatic reduction in Neurofilament 200 (Nf200) expression by neurons (Supporting Figure S11).

The possible functional impairment of microglial cells and astrocytes following the systemic administration of tPA was further evaluated in vivo on histological brain sections. The two markers for microglial cells (CD11b or Cluster of Differentiation 11b, a membrane marker) and astrocytes (GFAP marker) were spatially identified via immunostaining, and variations in morphology or protein expression were assessed. For the microglial cells, a clear activation was observed in the ipsilateral (Ip) hemisphere, where the CD11b signal (green fluorescence) was indicative of an activated branch-like microglial morphology as opposed to the most diffused distribution in the contralateral (Co) hemisphere (Figure 8A). The quantification of the area covered by the signal, as the number of activated cells, confirmed the differences between Co and Ip hemispheres but not among the three different treatments (Figure 8B). The microglial activation, documented by the augmented CD11b expression with the shrinkage of the cell body, was higher on the Ip compared to the Co hemisphere. GFAP staining also displayed a change in the expression of the marker between Co and Ip hemispheres (Supporting Figure S10A,B). The quantification of the area covered by the marker showed a similar GFAP expression level among the groups in the Co hemispheres. A significant increase of the expression in the Ip hemisphere for saline condition was observed (*p* = 0.0183); conversely, a nonstatistically significant increase of the tPA-DPN condition was observed (*p* = 0.0685) in comparison to the free-tPA (Figure 8D).

**Figure 8:**
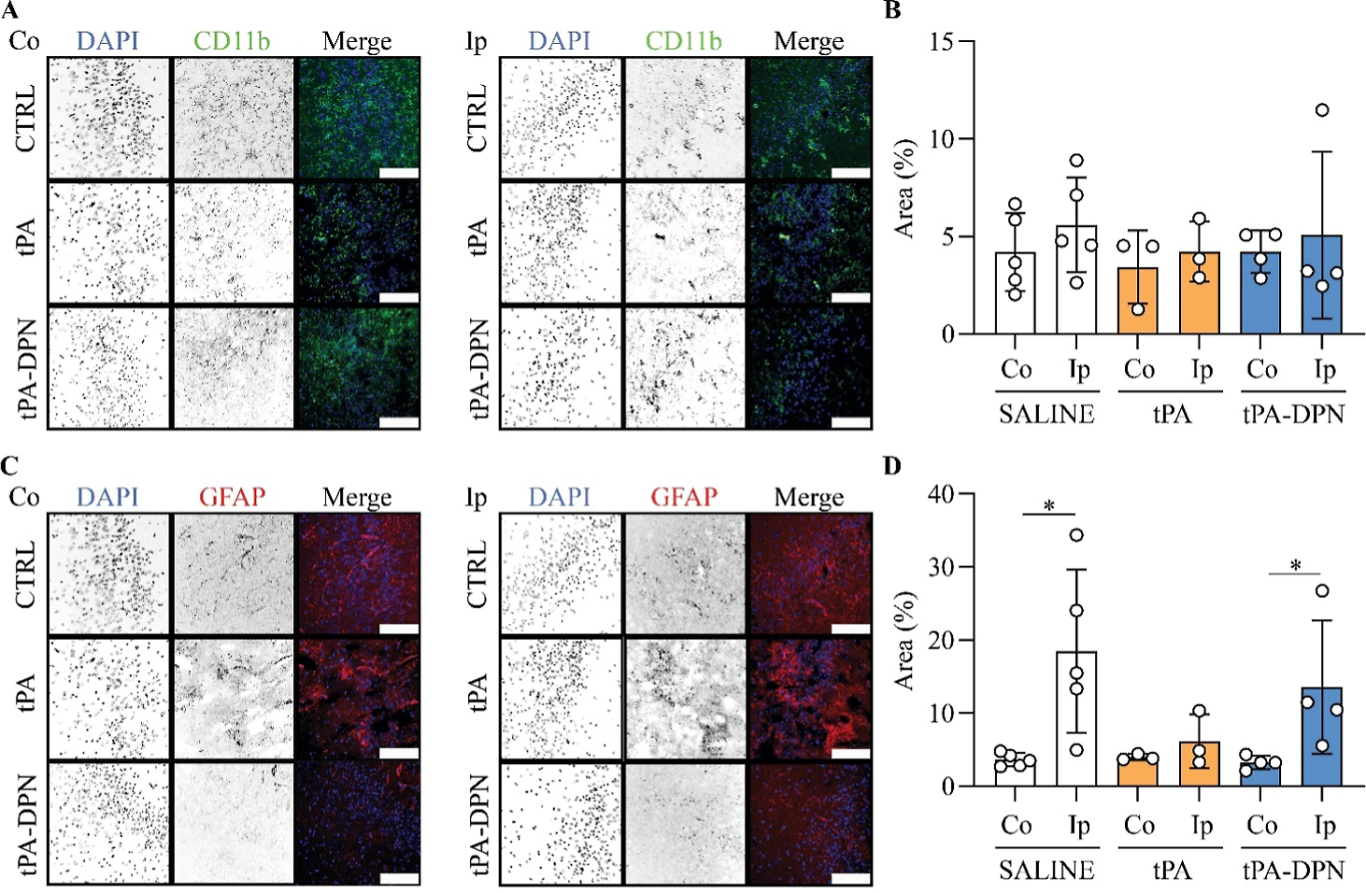
Immunofluorescence analysis of microglia and astrocytes response in stroke under the different treatments. (A) Representative images of microglial cells, stained for DAPI (nuclei, blue) and CD11b (green). The two panels represent the situation in the contralateral (Co) and ipsilateral (Ip) hemispheres. Black and white images of each row of the panel show the single marker expression for each area acquired, with the last color image displaying the composite image with all the markers. All images were acquired at 40× magnification (scale bar: 100 μm). (B) Percentage of area positive to CD11b expression, measured in Co and Ip hemispheres. Results are expressed as mean ± SD (*n* ≥ 3; two-tailed unpaired *t*-test). (C) Representative images of cerebral astrocytic cells, stained for DAPI (nuclei, blue) and GFAP (red). The two panels represent the situation in the contralateral (Co) and ipsilateral (Ip) hemispheres. Black and white images of each row of the panel show the single marker expression for each area acquired, with the last color image displaying the composite image with all the markers. All images were acquired at 40× magnification (scalebar 100 μm). (D) Percentage of area positive to GFAP expression, measured in Co and Ip hemispheres. Results are expressed as mean ± SD (*n* ≥ 3; *p < 0.05; two-tailed unpaired *t*-test).

Finally, the production of reactive oxygen species (ROS) is a major contributor to tissue damage in stroke, promoting neuroinflammation and vascular injury.^29^ Using the same procedures as those in the halo test shown in Figure 2, we assessed ROS production with the addition of the ROS probe 2′,7′-dichlorodihydrofluorescein diacetate (DCFH_2_-DA). Blood clots or whole blood were exposed to free tPA, tPA-DPN, DPN, and PBS as a control along with increasing concentrations of H_2_O_2_ (10 pM, 10 μM, and 10 mM). Supporting Figure S12 demonstrates that ROS were produced only in the presence of H_2_O_2_, in a dose-dependent manner, whereas neither tPA nor tPA-DPN contributed to ROS generation under these conditions. Furthermore, our in vitro and in vivo experiments revealed that tPA-DPN administration did not increase GFAP expression over saline, in contrast to free tPA treatments, which suppressed GFAP expression (Figure 7). This suggests that tPA-DPN may facilitate reactive gliosis, a physiological countermeasure to various nervous system disorders, potentially limiting the progressive expansion of the ischemic penumbra.^30^

## Discussion

Even though tPA treatment is beneficial and potentially lifesaving, neurotoxicity following tPA accumulation in the brain parenchyma is still a relevant limitation to the broad, unconditional use of this molecule.^31^ In addition to the well-documented increased risks of intracranial hemorrhage, tPA triggers a series of molecular events, including excitotoxic neuron cell death and potentiates apoptosis on the ischemic brain endothelium.^26,32^ Indeed, tPA is most effective when administered within a narrow time window after the onset of symptoms, typically within 3 to 4.5 h for ischemic stroke.^3,4,6^ Delayed administration reduces its efficacy and increases the risk of complications. A plethora of nanoscale carriers, including liposomes, iron oxide nanoparticles, and polymeric nanoparticles, have been used to deliver tPA.^9^ However, if these particles disassemble and release tPA or diffuse through the hyperpermeable vascular walls, thereby increasing the extravascular concentration of tPA, they could exacerbate the damage caused by the stroke, as in the case of free tPA. In contrast, micron-sized carriers such as microbubbles,^14^ deformable microparticles,^16^ or even red blood cells,^33^ when tPA is stably anchored to their surface, could limit the neurotoxicity associated with tPA administration. DPN falls into this category of vascular carriers.

DPN is a well-characterized drug delivery platform, originally designed for the vascular delivery of multiple therapeutic agents for oncological and cardiovascular applications.^16,19,34–36^ The chemical composition of these particles allows them to be flexible, minimizing recognition and sequestration by the reticuloendothelial system, thereby increasing blood longevity.^36^ Additionally, DPN can marginate under flow due to their nonspherical shape, facilitating interactions with endothelial cells, atherosclerotic plaques, as well as blood clots.^34^ DPN have demonstrated a favorable safety profile, with no observed metabolic impairment in human umbilical vein endothelial cells (HUVECs)^16,34^ and no significant changes in inflammatory cytokines (IL-6, IL-10, TNF-α) or serum enzyme levels (AST, ALT, creatinine) in vivo.^34^ In a mouse orthotopic breast cancer model, repeated intravascular administration of DTXL-loaded DPN showed no significant acute toxicity, with negligible effects on body weight and behavior.^37^ The direct binding of tPA to the DPN surface through a conventional EDC/NHS reaction returns a high bioconjugation efficiency, minimizing the losses of this expensive therapeutic agent. The tPA remains securely attached to the particle surface without unintended release for at least 72 h.^16^ As previously documented by the authors,^16^ over 70% of the in vitro clot lytic activity is preserved even after 3 h of incubation of tPA-DPN with FBS. The robust association of tPA with DPN, combined with prolonged circulation time and enhanced stability,^34^ ensures the efficacy and safety of the proposed approach.

Having already demonstrated the thrombolytic activity of tPA-DPN in a mesenteric model of stroke,^16^ here, we focused on its neuroprotection and safety using a fMCAO mouse model, which accurately mimics brain damage after cerebral blood vessel occlusion and reperfusion. The fMCAO model is an ideal method for highlighting the side effects of the drug and comparing the safety profiles of tPA-DPN and free tPA, as it exacerbates stroke injury caused by tPA administration, enabling a more accurate evaluation of the effects of delayed tPA treatment. The absence of an actual clot permits the observation of side effects without confounding factors, such as the rates of clot formation and dissolution. Our experiments showed that survival rate, activity scoring, and neurological scoring of mice at 24 h post-occlusion were severely affected by free-tPA treatment. Most free-tPA treated mice did not survive until 1 day poststroke, and the few survivors presented extreme neurological deficits. This aligns with current clinical and preclinical studies, showing that tPA administration with a compromised blood–brain barrier exacerbates stroke-induced cerebral tissue damage.^38^ Conversely, tPA conjugation to vascular-confined particles mitigated side effects, as the particles’ morphology prevented the extravascular accumulation of tPA in the brain parenchyma. Survival rates and behavioral scores poststroke observed in mice treated with tPA-DPN were comparable to those seen after saline administration.

These observations were further corroborated by histological analyses and magnetic resonance imaging data, which revealed smaller ischemic areas and a lower BBB permeability in mice receiving tPA-DPN as opposed to free-tPA. The results with tPA-DPN were similar to those obtained with saline injection. Such macroscopic tissue-level observations were also in agreement with cell-level analyses conducted on microglia and astrocytes. Astrocytes, an important component of the neurovascular unit, regulate the BBB permeability.^25,26^ During an ischemic event, endogenous tPA is secreted by perivascular astrocytes to increase local permeability. Exogenous tPA administered as a thrombolytic agent can cross the already impaired BBB, accumulate in the brain parenchyma, cause excitotoxic damage, and further increase the BBB opening. Indeed, MR imaging confirmed that free-tPA administration increased vascular permeability, lesion size, and brain damage, unlike that with tPA-DPN.

These results suggest that the strategy of firmly linking tPA to a micrometer particle reduces neurotoxicity and enhances safety. Unlike freely administered tPA, these particles do not extravasate at sites of vascular hyperpermeability following clot resolution. This strategy reduces the extravascular concentration of exogenous tPA, decreases brain cell damage, and improves prognosis, survival, and behavioral scores, as schematically summarized in Figure 9.

**Figure 9:**
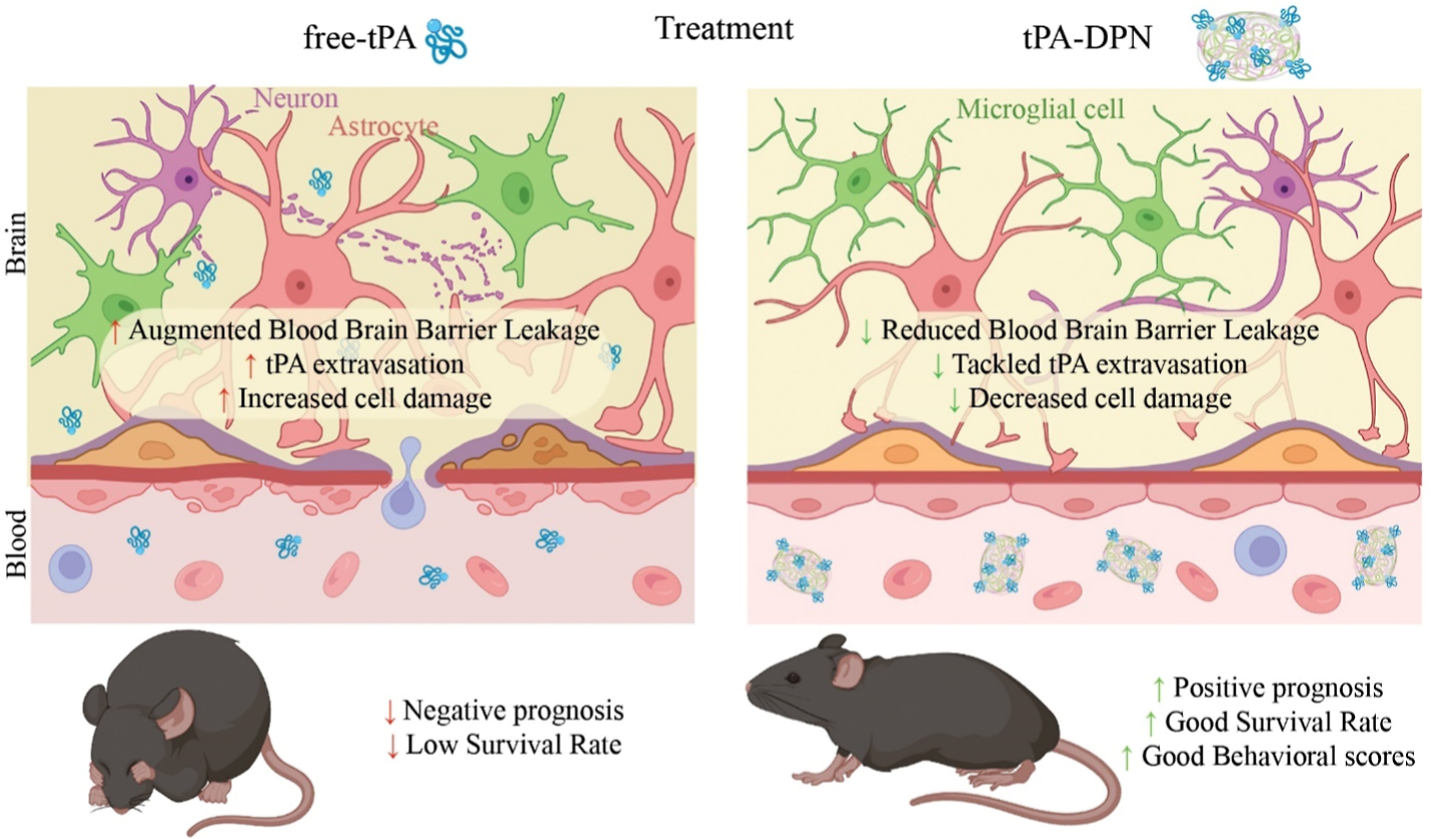
Advantages of using micrometric-particles as thrombolytic agents. The intravascular confinement of tPA using tPA-microparticles, like the tPA-DPN, reduces cerebral side effects and improves survival and behavioral outcomes.

## Conclusions

Spatial drug confinement inside blood vessels is a promising approach to reduce the side effects of systemic therapies, particularly in the case of thrombolytics administration for stroke, thereby extending the therapeutic window of this pharmacological treatment. In this context, tPA-DPN has been demonstrated to be an effective nanotechnology capable of mitigating the clinically documented side effects associated with the intravenous administration of tPA in the postacute ischemic stroke phase. tPA-DPN enhances the safety and therapeutic efficacy of tPA by preventing its uncontrolled accumulation within the brain parenchyma across an already damaged blood–brain barrier, boosting its blood longevity and protecting it from rapid loss of thrombolytic activity. The proposed direct conjugation of tPA to the surface of the vascular-confined discoidal polymeric nanoconstructs is anticipated to widen the temporal window for safe and effective tPA treatment.

Future studies should evaluate the proposed strategy across different stroke models, such as those induced by local administration of ferric chloride or thrombin, which vary in the severity of occlusions. In models with lower severity, combining histopathological analysis, live imaging, and behavioral evaluations would provide a more comprehensive understanding. Additionally, given the size of the microparticles and their versatile chemistry, investigating the combination of new thrombolytic agents such as Tenecteplase with neuroprotective molecules could further enhance both therapeutic efficacy and safety.

## Methods

### Reagents and Materials

Polydimethylsiloxane (PDMS, Sylgard 184) and elastomer were purchased from Dow Coming Corp. (Midland, US). Poly­(vinyl alcohol) (PVA, Mw 9000–10,000), poly­(d,l-lactide-*co*-glycolide) acid (PLGA, Resomer RG504 H lactide/glycolide 50:50, Mw 38,000–54,000), poly­(ethylene glycol) diacrylate (PEGDA, Mn 750), 2-hydroxy-40-(2-hydroxyethoxy)-2-methylpropiophenone (photoinitiator), 1-ethyl-3-(3-(dimethylamino)­propyl)-carbodiimide (EDC), *N*-hydroxysuccinimide (NHS), 2-hydroxy-4′-(2-hydroxyethoxy)-2-methylpropiophenone (Irgacure 2959, product code 410896), dichloromethane (Sigma-Aldrich, product code 270997), chloroform (Sigma-Aldrich, product code 319988) Certistain Cresyl Violet acetate for microscopy (Sigma-Aldrich, product code 105235), sucrose (Sigma-Aldrich, product code S7903), bovine thrombin (Sigma-Aldrich, product code 605157), deoxyribonuclease I from bovine pancreas (DNase) (Sigma-Aldrich, product code D5025–150 KU), Thiazolyl Blue tetrazolium bromide, 98% (Sigma-Aldrich, product code M2128–1G), Triton X-100 (Sigma-Aldrich X100), poly- *d* -lysine hydrobromide (PDL) (Merck Life Science, product code P6407–5MG), and bovine serum albumin (BSA) (product code A9418) were purchased from Merck (Merck KGaA, Darmstadt, DE).

Quantum Protein Bicinchoninic Protein Assay Kit (product code EMP014250) was obtained from EuroClone SPA (Pero, Milano, IT). Isoflurane was purchased from La Zootecnica Group SRL (Verrua Po, Pavia, IT) under a veterinary prescription. Paraformaldehyde (PFA) 0.4% solution in PBS was purchased from Santa Cruz Biotechnology Inc. (Heidelberg, DE). Surgipath FSC 22 Frozen Section Compound was purchased from Leica Microsystems GmbH (Wetzlar, DE). Rat IgG2b Anti-CD11b (eBioscience, product code 14-0112-82), rabbit IgG anti-GFAP (Invitrogen, PA1-10019), rabbit IgG anti-Iba1 (Invitrogen, product code PA5-27436), Permount Mounting Medium, and (Fisher Chemicals) ProLong Gold Antifade Mountant and ProLong Diamond with DAPI Antifade Mountant were from Thermo Fisher Scientific (Waltham, MA, US). Alexa Fluor 488 Goat Anti-Mouse IgG H&L (product code ab150113), Alexa Fluor 647 Goat Anti-Chicken IgY H&L (product code ab150171), and Chicken anti-GFAP (product code ab4674) were purchased by Abcam (Cambridge, UK). Vacutest cuvette for blood sampling was purchased from Kima SRL (Arzergrande, Padua, IT). Cell strainer 70 μm (ClearLine, product code 141349C) and UltraClean Closure 9 mm (product code 9003451) with Sterlitech disposable polycarbonate (PCTE) 2 μm membrane filters were obtained from Sterlitech (Auburn, WA, US, product code PCT2025100). All the reagents and other solvents were used without further purification.

ClearLine sterile cell strainer 40 μm was from dDBioLab (Dutscher group, ES). 96-well plates for the HALO test were obtained from Costar, Fisher Scientific (Thermo Fisher Scientific, Waltham, MA, US). Hanks’ balanced salt solution (HBSS) (product code 1417 5095), Trypsin (0.25%), phenol red (product code 25050014), Trypsin–EDTA (0.25%), phenol red (product code 25200056), DMEM/F-12 (Dulbecco’s Modified Eagle Medium/Nutrient Mixture F-12), GlutaMAX supplement (product code 31331028), HI Horse Serum heat inactivated (HS) (26050088), Penicillin–Streptomycin PEN-STREP (10,000 U/mL) solution (product code 15140122), Trypan Blue Solution 0.4% (product code 15250061), and Dulbecco’s phosphate-buffered saline (DPBS), no calcium, no magnesium (product code 14190144) were purchased from Gibco Fisher Scientific (Thermo Fisher Scientific, Waltham, MA, US).

Alteplase, a human-tissue-type plasminogen activator (tPA), was provided by the Policlinico San Martino Hospital in Genoa (IT).

Silicon filament (7-0 Max MCAO suture Re 2309, product code 70CL9TD023Re) and catheters (PI-191 microcatheter 150 mm connected to female Luer, product code PI-FL-191-152-150) were purchased from Doccol Corporation (Sharon, MA, US). Multisizer 4e Coulter Counter, Accuvette ST Sampling Vials (product code A35471), and ISOTON II Diluent were from Beckman Coulter (Cassina De’ Pecchi, Milan, IT).

GraphPad Prism was obtained from GraphPad Software (San Diego, California, US). All schematics were created with BioRender Software; some illustrations were adapted from SMART–Servier Medical Art Web site https://smart.servier.com/.

### Synthesis of tPA-Conjugated DPN

DPN were synthesized as described previously.^19^ Briefly, we followed a top–down approach: a silicon master template was fabricated using a laser writer lithographic technique returning a template with a specific geometrical pattern including an array of wells with a 1000 nm diameter and 400 nm height, nominally. This master silicon template was covered with a solution of PDMS (Sylgard 184, 10-parts base elastomer and 1-part curing agent), polymerized in oven at 60 °C for 4 h (h), returning a “positive” replica of the silicon template. Then, the PDMS template was covered with a PVA solution (5%) w/v and moved in the oven at 60 °C for 3 h to obtain a sacrificial PVA negative template identical to the original silicon master template. The water-soluble PVA templates were eventually used for the synthesis and purification of DPN. The original DPN polymeric mixture was prepared with PLGA (Resomer RG504 H, acid-terminated lactide/glycolide 50:50, *M*_w_ 38,000–54,000, 50 mg mL^–1^), PEGDA (*M*_n_ 750, 10 mg/mL), the photoinitiator Irgacure 2959 (1 mg mL^–1^), all dissolved in a mix of dichloromethane and chloroform (1:1 v/v). This solution was spread over the PVA template to carefully fill each single discoidal well and exposed to UV-light for 10 min (366 nm wavelength). For the particle collection, the so-loaded PVA templates were dissolved in deionized water under stirring conditions for 3 h at room temperature. The resulting aqueous suspension containing the DPN was filtered to remove residual debris through a 70 μm cell strainer (ClearLine), then centrifuged at 3200 rcf, 20 min, 4 °C. Purified particles were resuspended with deionized water, filtered by 2 μm filters (Sterlitech), and centrifuged twice at 18,200 rcf, 20 min, 4 °C.

For the conjugation of tPA, we followed the instructions described by Colasuonno et al.^16^ DPN were incubated with EDC and NHS, in a specific molar ratio with PLGA (EDC/NHS/PLGA 3:1), for 5 h under rotation at room temperature. To remove unreacted EDC/NHS, DPN were washed twice with deionized water and centrifuged at 18,200 rcf, 20 min, 4 °C. Activated DPN were then incubated overnight with Alteplase (10 μg tPA per mg PLGA). At the end of the conjugation step, the unbound tPA was removed via three washing steps with deionized water, followed by centrifugation at 18,200 rcf, 20 min, 4 °C.

### Physico-Chemical Characterization of DPN

DPN were characterized for yielding and geometry after purification and after conjugation with tPA for each batch of particles. Specifically, DPN were suspended in distilled water (1 mL water each 30 mg PLGA) and sonicated for 30 s before analysis. To evaluate the yield, we calculated the particle concentration with the Multisizer 4E Coulter Particle Counter (Beckman Coulter). For the analysis, an aliquot of this suspension (10 μL) was transferred to a Multisizer Cuvette with 20 mL of ISOTON II Diluent (Beckman Coulter). The measurements were run in technical triplicate using a 20 μm aperture Multisizer Capillar. DPN hydrodynamic size and surface electrostatic charge (ζ-potential) were measured by a Zetasizer Nano ZS (Malvern Instruments, Worcestershire, UK). Hydrodynamic size and surface charge were measured with automatic settings by the instrument (number of measurements, attenuator, and optimal measurement position). Each measure was done with at least three consecutive measurements and 11 runs per measurement. Further evaluation on the characteristic discoidal shape of DPN was confirmed by scanning electron microscopy (SEM, high resolution analytical field-emission scanning electron microscope) with JEOL JSM-7500FA, JEOL JSM-6490LA, and JEOL JEM-1011 equipped with a cold field emission gun (Jeol Ltd., Akishima, JP). Lastly, BCA assay was performed to determine the amount of tPA conjugated on the particles and the bioconjugation efficiency (BE), calculated as

1 $$BE\;\%=\frac{{\left[tPA\right]}_{final}}{{\left[tPA\right]}_{input}}100$$

### In Vitro Halo Clot Thrombolytic Assay

To evaluate the in vitro thrombolytic efficacy of tPA-DPN as compared to that of free-tPA, we used a modified version of the halo clot thrombolytic assay.^20^ All of the experiments were conducted on a Costar 96-well plate, as described. Rat blood was collected via hearth puncture into Vacutest tube (Vacutest Kima), with buffer citrate 3.2% as anticoagulant. Clot formation was induced with bovine thrombin (50 U/well), and 6.6 μL of this mixture was deposited at the bottom of the wells. Halo-shaped preclots were formed on the bottom of the wells by the addition of 10 μL of blood for each well. To obtain the clot formation, the plate was incubated at 37 °C for 30 min until the beginning of the test. The test was performed for 2 h at 37 °C. As positive control (100% clot lysis), a series of wells were prepared replacing bovine thrombin with Milli-Q water (no clot formation). As a negative control (0% clot lysis), clots were treated with saline solution (final volume considering a clot volume equal to 200 μL). Free-tPA or tPA-DPN were diluted into saline solution and 3 μg of each treatment was added in each well (final volume considering a clot volume equal to 200 μL, concentration of 214.3 nM). Each condition was tested at least in technical quadruplicate. Right after the addition of all the treatments, clot degradation was followed by the absorbance change at 510 nm every 2 min, using a Tecan Spark (Tecan, Männedorf, CH). The positive and negative control wells provided absorbance values corresponding to full clot lysis (*A*_total_) and no lysis (*A*_zero_), respectively. The percentage of clot dissolution (CD) over time was calculated as

2 $$CD\;\%\;\left(t\right)=\frac{\left(A_{x}\left(t\right)-A_{zero}\left(t\right)\right)}{\left(A_{total}\left(t\right)-A_{zero}\left(t\right)\right)}100$$

In addition to CD %, the following quantities were also estimated: the maximum clot lysis rate (CLR_max_), which corresponds to the maximum positive slope value of the degradation profile and describes the rate at which each treatment dissolves blood clots.; the time at which the slope reaches its maximum (*T*_CLRmax_); the activation time (*A*_(*t*)_), which represents the time (in min) when the slope of the degradation profile exceeds 1 for the first time, providing an indication of how rapidly the lysis process begins; and *T*_0.5_, which corresponds to the time needed to reach 50% lysis.

### Filament Middle Cerebral Artery Occlusion Stroke Model

All animal procedures were performed following the Italian laws and under the approval of the internal Ethical Committee for Animal Experimentations (OPBA) and the Italian Ministry of Health (animal protocol 176AA.54, ID 156/2019 PR, approved on 25 February 2019). The animal model selected for this study is one of the most extensively described and used preclinical murine models available for stroke.^39^ Surgical procedures were conducted according to the method of Koizumi.^40^ The model is generated by a transient occlusion of the middle cerebral artery (MCA) realized by a silicon filament (fMCAO), on 8- to 12 week-old wild-type C57BL/6J male mice. For the last 3 days before surgery, the weight of the animals was registered daily to monitor their health status. On the day of the surgery, all the animals were tested for activity and neuroscore, which had to be all negatives (absence of behavioral pre-existing deficiency). Considering this evaluation, no animals were discarded. After behavioral assessment, all of the animals were shaved around the ventral side of the neck and breast. For the surgery, animals were anesthetized with isoflurane (4% for induction and 1.5–2% for maintenance). During surgery, body temperature was monitored (RWD ThermoStar Homeothermic Monitoring System, RWD Life Science Co.,LTD, Shenzhen, CN) as well as the breathing and heart rate (MouseOx Plus Pulse Oximeter with mouse paw sensor, STARR Life Sciences Corp., Oakmont, PA, US). With the animal supine, after disinfection with a betadine solution (Meda Pharma S.P.A), an incision was made in the neck area. To highlight the left common carotid artery (CCA), soft and muscle tissues were gently stretched apart. Once the branching between CCA, the internal carotid artery (ICA), and the external carotid artery (ECA) was identified, one ligature with a sterile thread (F.S.T. 5–0 Nonabsorbable Braided Silk Suture) was applied on ICA and ECA, and two ligatures were applied to the CCA, one distal and one more proximal to the branching, to block the blood flow. By a hole created at the bifurcation site, a silicon filament (Doccol 7–0 Max MCAO suture) was inserted through the ICA and pushed inside the vessel until it reached the middle cerebral artery (MCA). The success of the occlusion was checked in real-time by assessing local blood flow variations via a dedicated instrument (Laser Doppler Blood FlowMeter No. INL191, with a Laser Doppler Needle OxyFlo Probe, MNP100XP-3/10, ADInstruments Ltd., Oxford, UK). Then, the proximal ligature on the CCA was tightened to fix the filament with the occlusion lasting for 60 min. The animal was awakened from anesthesia and transferred to a clean cage under the heating lamp. After 60 min, the filament was removed and the ligatures were tightened. In the SHAM groups, all of these manipulations were performed, but the filament was removed just immediately after reaching the MCA. Twenty minutes after filament removal, we performed the intravenous administration of the treatments (10% bolus, 90% infusion over 20 min of saline, tPA 10 mg kg^–1^, tPA-DPN 10 mg kg^–1^) by a syringe pump (Pump 11 Elite, Harvard Apparatus, Holliston, MA, US). In vivo experiments were conducted using a simple randomization approach and an open-label blinding method. Note that a tPA dose of 10 mg·kg^–1^ is commonly used in small rodents’ experiments to demonstrate efficacy, which corresponds to the human equivalent dose (HED) of 0.9 mg·kg^–1^, currently used in the clinical setting.^23,41–45^ The number of particles and the amount of tPA associated with tPA-DPN were determined using the Multisizer coulter counter and analytical techniques (BCA assay), respectively, and as described above. Notably, the particle concentration used in this work is well tolerated by mice and did not show any signs of acute or subacute toxicity. These observations are consistent with previous studies.^16,34,37^ The administration was performed through the tail vein using a microcatheter, connected to a syringe pump for the controlled infusion of the treatments. At the end of the procedures, animals were transferred back to their original cage and kept under a heating lamp until awakening.

### Activity and Neurological Severity Scoring

Behavioral assessments were made before the surgery to exclude animals with pre-existing behavioral deficits and 24 h post-occlusion. Activity scoring was calculated from data collected by the MouseOx Plus instrument using the collar sensor. The recordings of the movements of the animals were performed every 2 s for at least 5 min. The frequency of the movements of the animal, scored as 1, was divided by the number of total measurements to obtain the “Activity Score”. Differently, the behavior of the animals was measured by Neurological Severity Scoring (NSS), modified from Jiang et al. as follows: score 0: normal; score 1: mild turning behavior, with or without inconsistent curling when picked up by tail, and 50% attempts to curl to the contralateral side; score 2: mild consistent curling, 50% attempts to curl to contralateral side; score 3: strong and immediate consistent curling, mouse holds curled position for more than 1–2 s, the nose of the mouse almost reaches tail; score 4: severe curling progressing into barreling, loss of walking or righting reflex; score 5, comatose or moribund; score 6: found dead.^46^

### Magnetic Resonance Imaging Analysis

Experiments were performed on a 7T MRS* PET-CO 803 system (MRSolutions, UK). Animal breath rate was monitored with an abdominal pillow working as a pressure sensor, and all data were recorded with PC Sam software (SAII, Stony Brook). For the *T*_1_ weighted image acquisition (*T*_1w_), a fast spin echo sequence was adopted, with echo time (TE) of 11 ms and repetition time (TR) of 1000 ms. For *T*_2_ weighted imaging (*T*_2w_), TE and TR were set to 45 and 3000 ms, respectively. *T*_1w_ and *T*_2w_ images were acquired in the axial plane with a 1 mm slice thickness. To perform the experiments on BBB leakage, 50 μL of a 0.5 M solution of ProHance (Bracco Imaging Italia SRL, Milano, IT) was injected as a contrast agent, 15 min before starting the measurements. Whole brain and lesion volumes were quantified with Fiji software.^47^

### Mixed Glial Cell Culture Preparation

Mixed glial cell cultures were prepared from C57BL/6J mice at P1–P2.^48^ After the pups were sacrificed by dislocation, all of the steps were performed in Petri dishes with ice-cold HBSS and sitting on ice. The skull and cerebral dura mater were removed, and brains were extracted from the skull and transferred into a new dish. Meninges were pulled out, and cortices were dissected from the brain and placed in cold HBSS. 3–4 brains were moved into a 15 mL sterile tube containing 3–4 mL of 0.25% trypsin in HBSS and 500 μL of DNase I stock. Deoxyribonuclease I from bovine pancreas was prepared as stock at 25 mg/mL in HBSS and stored at −20 °C. The tissue was incubated in water bath at 37 °C for 15 min and mixed gently turning the tube every 5 min. At the end of the process, 10 mL of complete glial cell medium (DMEM/F12-GlutaMAX, 10% HS and 1% PS solution) was added to stop the digestion. The cells were centrifuged for 5 min at 270 g, the supernatant was carefully removed, and 3–4 mL of fresh glial cell media was added. The tissue was then dissociated into a single cell suspension pipet 10 times. The dissociated cells were passed through a 40 μm strainer and placed onto a 50 mL centrifuge tube to filter out any remaining tissue fragments. Live cells were counted in a hemacytometer by mixing 10 μL of cell suspension with 10 μL of 0.4% Trypan blue, and then the appropriate volume of glial cell media was added to get the desired cell density. We plated the cells on PDL (0.1 mg/mL) coated 96-well plates at a density of 100,000 cells/cm^2^ (30,000 cells/well) in 200 μL or coated 24-well plates at a density of 50,000 cells/cm^2^ (100,000 cells/well) in 500 μL of media or at a density of 2–2.5 × 10^6^ cells in 10 mL of media in 75 cm^2^ (T75) flasks. Cells were grown at 37 °C in a humidified atmosphere of 5% CO_2_. After 1 day, the total volume of media was refreshed and exchanged with fresh media to remove debris and unattached cells, and afterward every 6–7 days, half of the volume was exchanged with fresh media maintained in culture for up to 14–21 days (DIV14-DIV21) before starting experiments. Cells were treated for 24 h with different concentrations of free-tPA, tPA-DPN, and corresponding amount of DPN. Cell morphology, immunofluorescence analysis with signal intensity quantification, and viability assay were performed.

### Microglial Primary Cells Isolation

Microglial cells were selected using the shaking method described in Giulian and Baker, 1986.^49^ Cells extracted from mice cortices were plated on PDL-coated 75 cm^2^ culture flasks and grown at 37 °C in a humidified atmosphere of 5% CO_2_ in glial cell media. After 14 days, microglial cells were obtained by shaking the flasks for 3 h at 250 rpm. Floating cells were removed, pelleted at 270 rcf for 5 min, and subcultured at 200,000 cells/cm^2^ (60,000 cells/well) in 96-well plates with conditioned medium. Cells were treated for 24 h with different concentrations of free tPA and then viability assay was performed.

### Astrocyte Primary Cells Isolation

Following the shaking step needed to isolate microglial cells, the 75 cm^2^ culture flasks were rinsed twice with DPBS, and 5 mL of 0.25% trypsin–EDTA was added. The flasks were placed in the incubator at 37 °C, and after 30 min, the cell detachment was enforced by hitting the flask bottom with the hand a few times. After astrocytes detachment, 5 mL of complete glial cell media were added to block trypsin activity and collect the cells. The media containing cells were spun at 270 rcf for 5 min, and the supernatant aspirated. The cells were resuspended in fresh preheat media, and living cells were counted and seeded to have a cell density of 100,000 or 200,000 cells/cm^2^ (30,000 or 60,000 cells/well) in 96-well plate.^48^ Cells were maintained at 37 °C in the CO_2_ incubator for 4 days, and then cells were treated for 24 h with different concentrations of free tPA and viability assay was performed.

### Hyperpermeable BBB Transwell Experiments with Mixed Glial Cultures

Mixed glial cultures were prepared as described above. After mechanical and chemical dissociation, cortical cells were seeded in 24-well plates at 50,000 cells/cm^2^ (100,000 cells/well) in glial cell media and cultured at 37 °C in humidified 5% CO_2_ for 7 days. Medium was replaced completely with fresh media after 1 day and afterward every 4 days. Cells were treated for 24 h with different concentrations of free-tPA (150 μg/mL), tPA-DPN (150 μg/mL), and corresponding amount of DPN. Cell morphology, immunofluorescence analysis with signal intensity quantification, and viability assay were performed.

### Cell Metabolism Analysis

An MTT assay is a colorimetric assay that detects the color change from yellow of the thiazolyl blue tetrazolium bromide dye to purple due to the formation of formazan in the presence of viable cells with active metabolism. Mixed glial cells, purified microglial cells, and astrocytes were cultured in 96-well plates as described above and maintained at 37 °C in 5% CO_2_, in DMEM/F-12, GlutaMAX medium supplemented with 10% HS, 1% PS. Cells were treated with different concentrations of free-tPA (namely, 1, 10, 40, 80, 160, 200, 240, 280, 320, 400 μg·mL^–1^) or an equivalent number of empty DPN matching the different tPA-DPN concentrations. At the end of the designated incubation times, 5 mg mL^–1^ of MTT solution in DPBS was added to each well, and the cells were incubated for 4 h at 37 °C. The solubilized formazan product was dissolved with absolute alcohol (100–200 μL/well) and quantified using a spectrophotometer at 570 nm using 650 nm as the reference wavelength (Tecan, Männedorf, Swiss). The percentage of cell metabolism was assessed according to the following equation

3 $$cell\;metabolism\;\left(\%\right)=\frac{{Abs}_{t}}{{Abs}_{c}}100$$

where Abs_t_ and Abs_c_ are the absorbance of treated cells and untreated (control) cells, respectively.

### Immunofluorescence on Primary Cell Cultures

Mixed glia and purified glial cells were both cultured on glass coverslips in 24-well plates as described above, before fixation with 4% PFA. To perform the staining, cells were incubated 4 h at room temperature with the blocking solution (2% BSA, 0.1% Triton X-100 in DPBS), followed by an overnight incubation at 4 °C with primary antibodies (chicken anti-GFAP, dilution 1:700 v/v; rabbit anti-Iba1, dilution 1:500 v/v) in 1% BSA and 0.1% Triton X-100 solution. The cells were washed with DPBS three times and then incubated for 2 h at room temperature with secondary antibodies (Alexa Fluor 488-labeled goat antirabbit antibody, dilution 1:1000 v/v; Alexa Fluor 647-labeled goat antichicken antibody, dilution 1:1000 v/v). After three washes with DPBS, coverslips were mounted with ProLong Diamond Antifade Mountant with DAPI (Thermo Fisher Scientific) and stored at 4 °C until the analysis.

### Histology

Histological analysis was performed on brain samples collected from the animal at the experimental end point. After the brain dissection, the entire organ was transferred in fixative solution (4% PFA in PBS) for 24 h at 4 °C. Then, organs were washed once with PBS, and prepared for cryosectioning, by two incubation steps in 20% sucrose in PBS, followed by 30% sucrose in PBS, each step for at least 24 h (until sample sunk in the solution) at 4 °C. At the end of this process, samples were ready to be frozen with vapors of liquid nitrogen and finally transferred at −80 °C, until sectioning. Prior to sectioning, each sample was embedded in Surgipath FSC 22 Frozen Section Compound. See Supporting Figure S4 for images depicting the approach used for the sectioning. Serial coronal sections of 20 μm thickness were prepared from each brain samples, to have every 1000 μm (1 mm) of the whole brain on each slide. Slides were stored at −80 °C until staining. For lesion volume quantification, we performed Cresyl Violet (CV) staining. CV staining solution was prepared at 0.1% w/v, dissolving the powder in water at 60 °C. Before the use, the staining solution was heated at 60 °C and filtered. Briefly, the sections were stained with CV staining solution for 10 min at 60 °C, then dehydrated in ethanol, cleared with xylene, and mounted in Permount Mounting Medium. For Immunoglobulin G (IgG) staining on the brain slices, sections were permeabilized with 0.25% Triton X-100 in DPBS for 15 min at room temperature, blocked with 5% BSA in 0.1% Triton X-100 in DPBS for 1 h at room temperature, and incubated for 2 h at room temperature in a humid chamber with Alexa Fluor 488 Goat Anti-Mouse antibody, diluted in 1% BSA, 0.1% Triton X-100 in DPBS (1:200 v/v). For nuclear counterstaining, sections were incubated with Hoechst 33342 for 30 min at room temperature (Hoechst 1:750 v/v, in 1% BSA in DPBS). Finally, sections were mounted with ProLong Gold Antifade Mountant (Thermo Fisher Scientific). Images were acquired at 4× magnification by an Olympus BX-51 upright microscope, with Optronic Microfire A/R camera (Olympus Lifescience, Milan, IT), driven by stage controller and Neurolucida software (MBF Bioscience & LUDL Electronic Products LTD, Hawthorne, NY, US). For glial cells staining on the brain slices, sections were permeabilized with 0.25% Triton X-100 in DPBS for 15 min at room temperature, blocked with 5% BSA in 0.1% Triton X-100 in DPBS for 1 h at room temperature, and incubated for 2 h at room temperature in a humid chamber with a specific primary antibody diluted in 1% BSA, 0.1% Triton X-100 in DPBS (eBioscience Rat IgG2b Anti-CD11b, 1:300 v/v; Invitrogen Rabbit IgG anti-GFAP, 1:500 v/v; Invitrogen Rabbit IgG anti-Iba1, 1:500 v/v; Abcam Chicken anti-GFAP, 1:1000 v/v). Then, sections were incubated with a secondary antibody (Alexa-Fluor 596 Goat anti-Rabbit 1:1000 v/v; Alexa Fluor 488 Goat anti Rat 1:500 v/v; Alexa-Fluor 647 Goat anti-Chicken 1:1000 v/v), in 1% BSA in DPBS, for 1 h at room temperature, in a humid chamber, and protected from light. For nuclear counterstaining, sections were incubated with Hoechst 33342 for 30 min at room temperature (Hoechst 1:750 v/v, in 1% BSA in DPBS). Images were captured by A1 Confocal Laser Scanning Microscope, at 40× magnification (Nikon, Tokyo, JP). Finally, sections were mounted with ProLong Gold Antifade Mountant (Thermo Fisher Scientific).

### Imaging Analyses

The extent of the infarcted region (lesion size) and the permeability of the BBB were measured through histological analyses and by postprocessing analysis of the MRI acquisition. The Fiji software was employed.^47^ For the lesion size, CV-stained histological sections from each sample were assembled in a single “stack” file. Stacks were converted to 8 bit files, returning grayscale images. Manual adjustment of the contrast and brightness was performed to highlight healthy and lesioned areas in every section. Characteristic volumes of the lesion and adjacent regions were calculated using the formulas

$$V_{i}^{les}=d\times A_{i}^{les};lesion\;volume$$

4 $$V_{i}^{Ip}=d\times A_{i}^{Ip};ipsilateral\;volume$$

$$V_{i}^{Co}=d\times A_{i}^{Co};\;controlateral\;volume$$

where A_*i*_^les^, A_*i*_^Ip^, and A_*i*_^Co^ are the area of the lesion and the ipsilateral and contralateral areas at the histological section *i*, respectively, with *d* being the distance between adjacent sections *i* and *i+*1. To exclude the impact of Edema from volumetric quantifications, we calculated the Edema ratio between the ipsilateral and the contralateral hemisphere as follows

5 $${OE}_{i}=V_{i}^{Ip}/V_{i}^{Co}$$

The total brain lesion volume V_TOT_^les^ derived from the sum of all the sections lesion volumes V_i_^les^ normalized against its section Edema Impact OE_*i*_, with the formula

6 $$V_{TOT}^{lesOE}=\sum_{i}^{n}V_{i}^{les}/{OE}_{i}$$

Edema Impact OE_*i*_ was also used to normalize the total brain volume V_TOT_^BrainOE^, obtained as follows

7 $$V_{TOT}^{Brain\;OE}=\sum_{i}^{n}\left(V_{i}^{Ip}/{OE}_{i}\right)+V_{i}^{Co}$$

Different regions of interest (ROI) were created on the images, selecting ipsilateral/contralateral hemispheres and lesioned area by the tool “Polygon Selections”. Every created ROI was saved in the “ROI Manager” in Fiji software (Menu Analyze > Tools > ROI Manager) for further analyses. After setting the scale bar (Menu Analyze > Set Scale), all the ROI areas were quantified (with ROI Manager, after selecting all the ROI, with the command “Measure”) and used for the volume calculations, as described by the formulas above.

The same process was followed for the image analysis after IgG staining (Immunofluorescence). In this case, RGB images for each section and fluorescent channel acquired with the microscope (FITC channel for IgG, DAPI channel for nuclei) were singularly converted into 8 bit images (grayscale). FITC and DAPI 8 bit images of each section were merged with the tool “Merge Channels” (Menu Image > Color > Merge Channels) to obtain a “Composite” 8 bit image with the 2 channels overlapped. All composite images from each sample were assembled in a single “Stack” file, by the command “Concatenate” (Menu Image > Stack > Tools > Concatenate). For the measurements and calculation, the procedure adopted is described above. For the lesion size quantification on MRI images, the method used was akin to the one adopted for the lesion size on CV-stained sections.

### Methodology for Volume Calculation and 3D Reconstruction

Source images, 2D slices of brain and stroke, and BBB leakage areas were obtained from widefield microscopy or MRI. To construct 3D images from the 2D data set (source images), a distance between adjacent slices (slice thickness *d* = 1 μm) and the size of 2D image in pixel were derived from the imaging devices. By stacking the images sequentially, 3*D* point clouds can be generated in point cloud file (ply). However, due to the relatively small number of images in a stack (7–9 slices) and large slice thickness, a method was proposed to generate additional images in-between slices, detect edges, extract contour points, and save them into a point cloud file. The algorithm for 3D reconstruction is detailed in the Supporting Information.

### Statistical Analysis

Data were expressed as mean ± standard deviation (SD), from at least three independent experimental units (experimental units defined as primary cell culture, tPA batches, or mouse, respectively). Where indicated, statistical analyses were performed using GraphPad Prism software. Specifically, the following tests were used: unpaired two-tailed Student’s *t*-test, one or two-way ANOVA variance test combined with the Tukey ad hoc correction method, or Log-rank (Mantel–Cox) test with Kaplan–Meier survival analysis (see Supporting Table S1)

## Supplementary Material



## Notes

In vivo experiments (fMCAO stroke model): additional data on survival rates and behavioral test scores in both treated and SHAM conditions; photographic documentation showing increased hemorrhage formation in the ischemic area following free-tPA treatment; histological and MRI analysis: detailed methodology for histological and MRI image analysis; comparative data on total brain volume and edema quantification across different experimental conditions; 3D volume reconstruction: description of the reconstruction methods and the algorithm used for 3D volume rendering; in vitro experiments: additional materials, methods, and results from experiments involving glial cells and neurons; data on Reactive Oxygen Species (ROS) production in the halo clot thrombolytic assay, tables summarizing statistical analyses, and references (PDF)

## Abbreviations

AS: activity score
BBB: blood brain barrier
BE: bioconjugation efficiency
CCA: common carotid artery
CD: clot dissolution
Co: contralateral
CV: Cresyl Violet
DIV: days in vitro
DPN: discoidal polymeric nanoconstructs microparticles
DTXL: docetaxel
ECA: external carotid artery
EDC: 1-ethyl-3-(3-(dimethylamino)­propyl)-carbodiimide
EE: encapsulation efficiency
fMCAO: filament middle cerebral artery occlusion model
HED: human equivalent dose
ICA: internal carotid artery
IgG: immunoglobulin G
Ip: ipsilateral
MCA: middle cerebral artery
MCAO: occlusion/reperfusion of the MCA
MRI: magnetic resonance imaging
NHS: *N*-hydroxysuccinimide
NSS: neurological severity score
PDMS: polydimethylsiloxane
PEG: poly­(ethylene glycol)
PEGDA: poly­(ethylene glycol) diacrylate
PLGA: poly­(d,l-lactide-*co*-glycolide acid)
PS: post stroke
PVA: poly­(vinyl alcohol)
SEM: scanning electron microscopy
tPA: recombinant tissue-type plasminogen activator
