# Supplementary material for: Enhancing Thrombolysis Safety in Post-Acute Ischemic Stroke with Tissue Plasminogen Activator-Associated Microparticles
Source: ACS Nano. 2025 Jun 11;19(25):22882–99. doi: 10.1021/acsnano.5c01499 (PMC12224294; doi:10.1021/acsnano.5c01499)
Supplement: Supplementary file 1 [file nn5c01499_si_001.pdf]

SUPPORTING INFORMATION

ENHANCING THROMBOLYSIS SAFETY IN POST-ACUTE ISCHEMIC STROKE WITH TISSUE PLASMINOGEN ACTIVATOR ASSOCIATED MICROPARTICLES

Raffaele Spanò [a], Corinne Portioli [a], Tijana Geroski [b], Alessia Felici [a], Anna Lisa Palange [a], Peter James Gawne [a], Stefania Mamberti [a], Greta Avancini [a], Roberto Palomba [a], Thomas Bonnard [c], Thomas Lee Moore [a], Massimo Del Sette [d], Nenad Filipovic [b], Denis Vivien [c, e], Paolo Decuzzi [a, f, *]

[a] Laboratory of Nanotechnology for Precision Medicine, Fondazione Istituto Italiano di Tecnologia, Genoa, 16163, Italy;

[b] Faculty of Engineering, University of Kragujevac, Kragujevac, 34000, Serbia;

[c] Physiopathology and Imaging of Neurological Disorders (PhIND), Normandie University, UNICAEN, INSERM, GIP Cyceron, Institute Blood and Brain @ Caen-Normandie (BB@C), Caen, 14000, France;

[d] Neurology Unit and Stroke Unit, IRCCS Ospedale Policlinico San Martino, Genoa, 16132, Italy;

[e] Clinical Research Department, CHU de Caen Normandy, Caen, 14000, France;

[f] Division of Oncology, Department of Medicine and Department of Pathology, Stanford University School of Medicine, Stanford, 94305, CA, USA.

[*] corresponding author: Paolo Decuzzi, PhD paolo.decuzzi@iit.it

Supporting Figures and Results

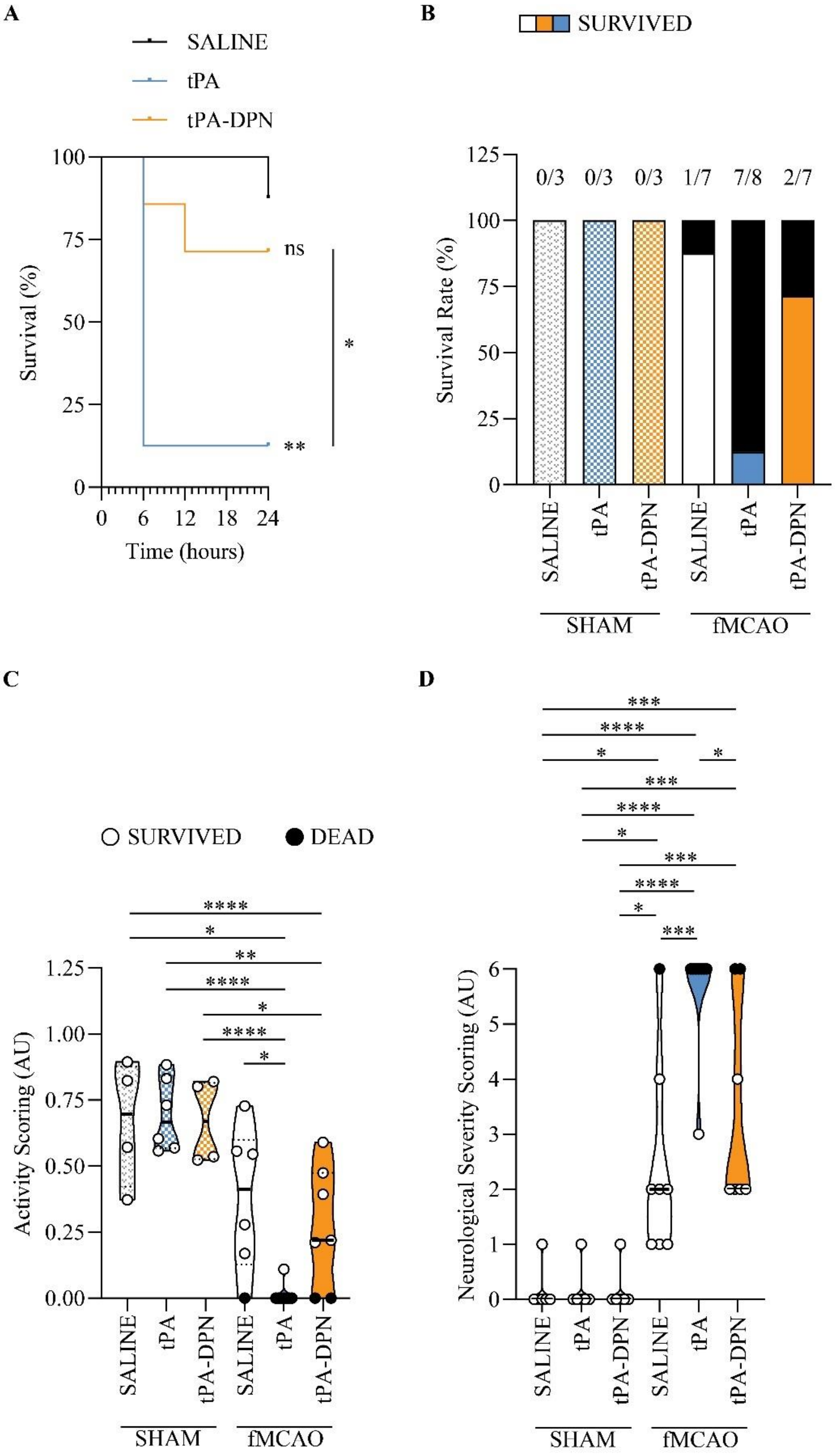

Supporting Figure 1: Survival rates and Behavioral tests. (A) Percentage of survival rate described by Kaplan-Meier curves. Log-rank (Mantel-Cox) on Kaplan-Meier curves tests show the statistical differences in average survival between all the groups ($p = 0.0019$), SALINE vs. tPA ($p = 0.0016$), tPA vs. tPA-DPN ($p = 0.0143$) but no differences between CTRL vs. tPA-DPN ($p = 0.3995$). **(B)** Percentage of survival rate between sham (no occlusion) and fMCAO groups. **(C)** Activity Scoring, and **(D)** Neurological Severity Scoring (NSS). Black symbols refer to animals found dead after 24h ($n \geq 4$; *$p < 0.05$, **$p < 0.01$, ***$p < 0.001$, **** $p < 0.0001$ respectively; one-way ANOVA, with Tukey correction).

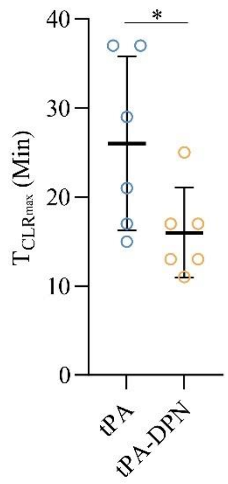

Supporting Figure 2: In vitro thrombolytic efficacy of tPA-DPN by halo assay. The graph shows the time (T) at which the clot degradation rate reaches its maximum (CLRmax) (n = 6, *p = 0.05, Two-tailed Unpaired t test).

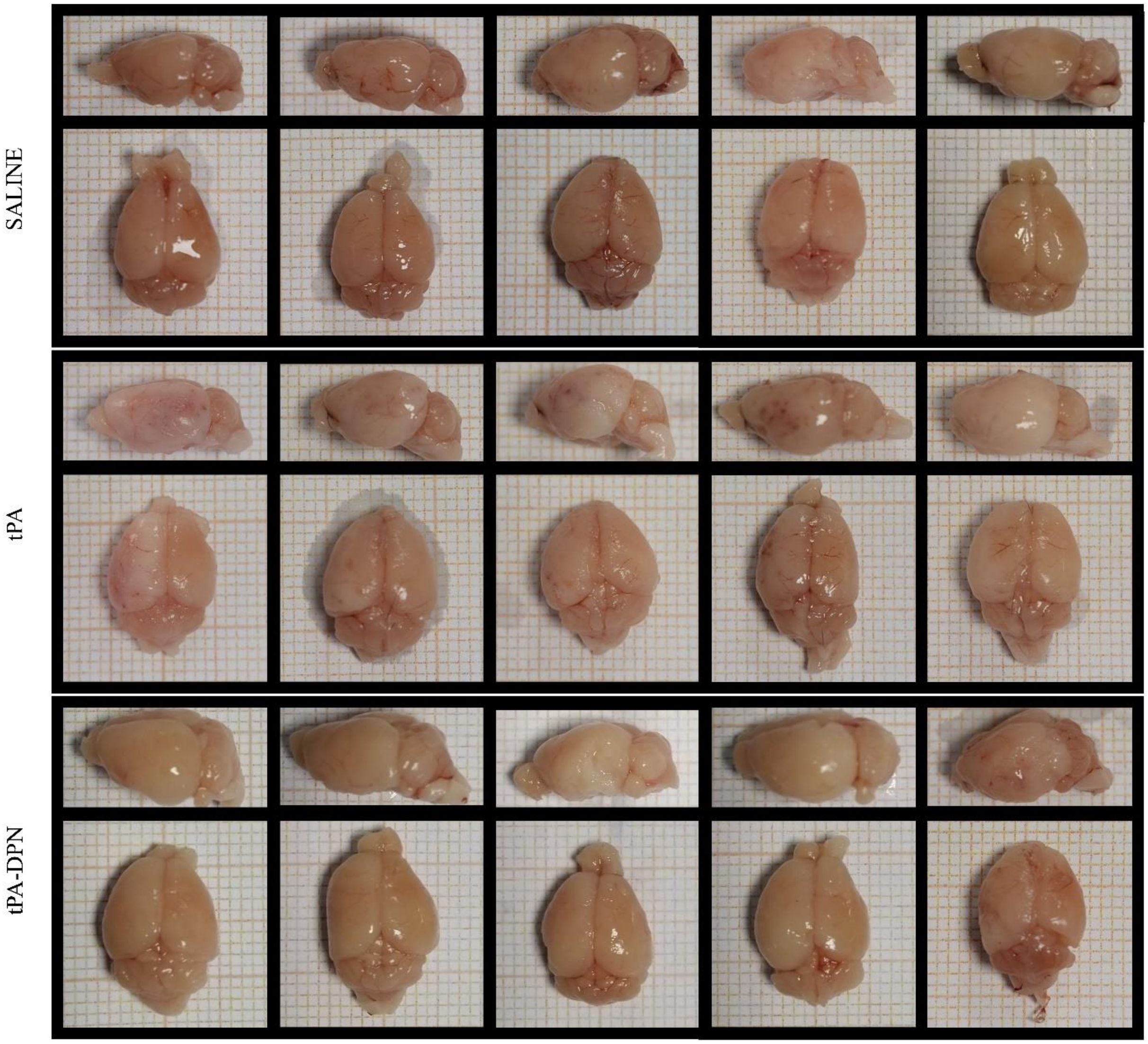

Supporting Figure 3: Free-tPA treatment increased hemorrhage formation in ischemic area. The panel shows representative photographic acquisitions of murine brain after stroke before histological analysis. In addition to a common increased volume of the left ipsilateral hemisphere, the left side lateral view of each brain displays an increased presence of hemorrhagic spots in the free-tPA treated group, in comparison to saline and tPA-DPN (graph paper scale: 1 mm).

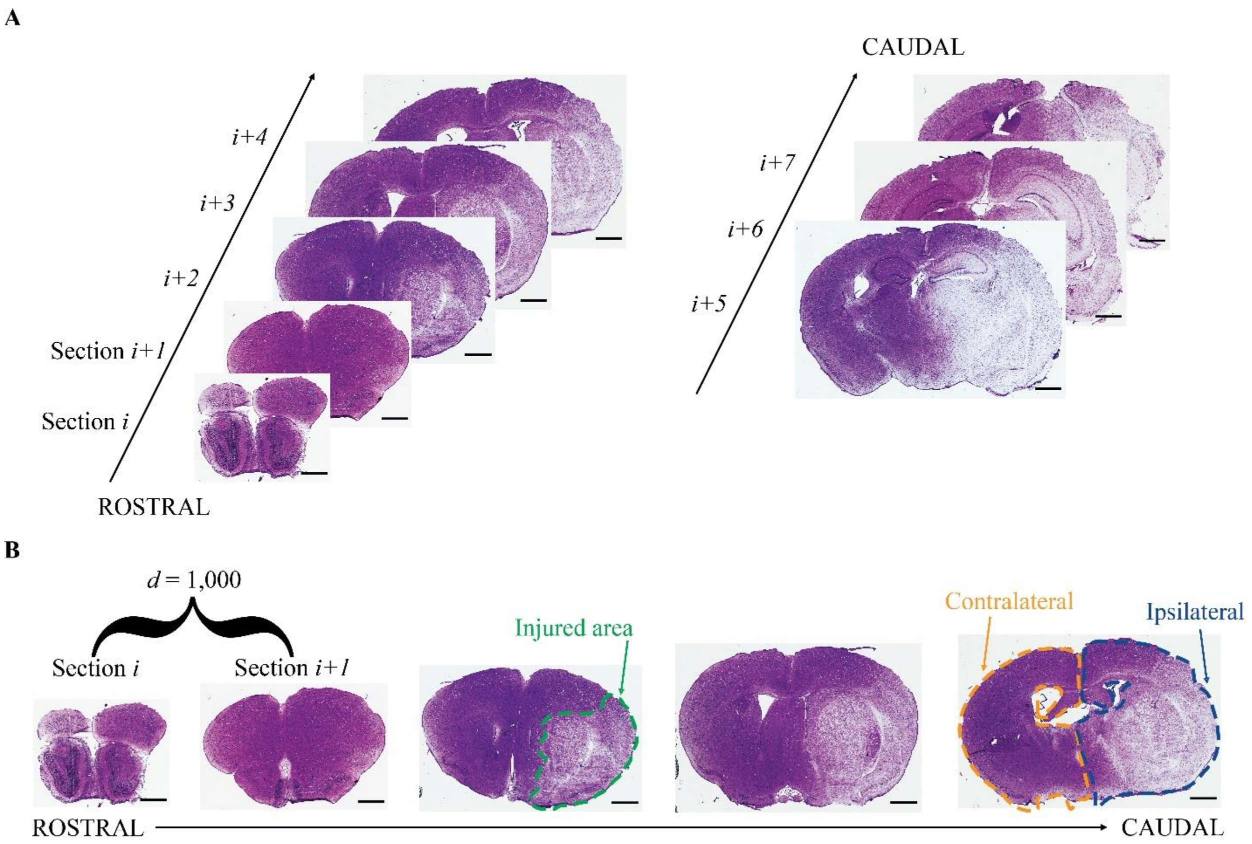

Supporting Figure 4: Imaging analyses. (A) The panel shows the series of coronal histological sections of a representative brain sample, collected from the rostral to the caudal part. **(B)** Distance among sections (*d*), as well as lesion area (A_i^{les}), contralateral and ipsilateral area ($A_i^{Co}$and A_i^{Ip}, respectively) are detailed. Scalebar, 1 mm.

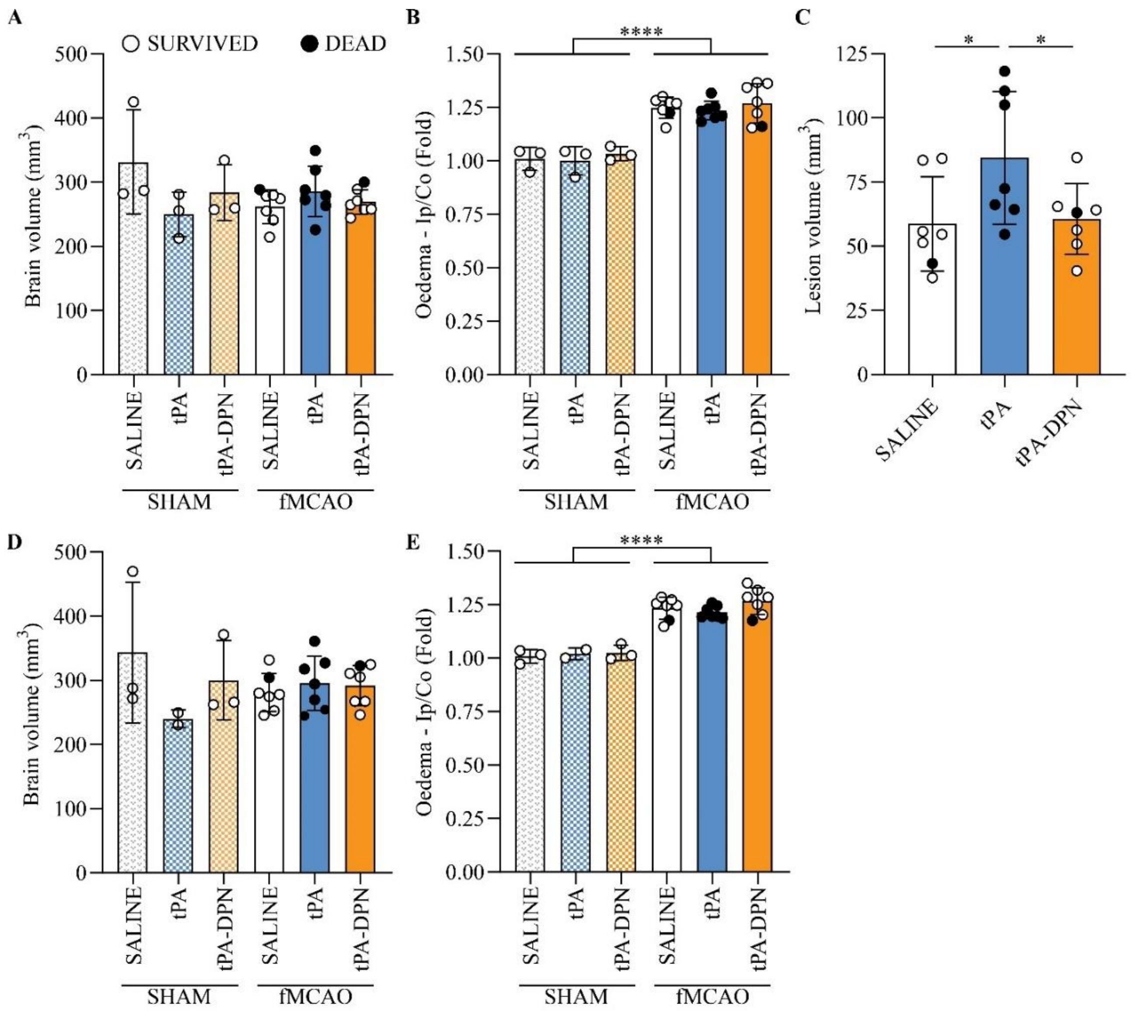

Supporting Figure 5: Comparison of total brain volume size and oedema quantification for the different tested conditions. (A) Whole brain volume measurements on the fMCAO group samples, after CV staining. Filled circles refer to mice found dead within the first 24h post-occlusion. **(B)** Oedema evaluation calculated from the CV-stained samples. **(C)** Whole lesion volume measurements on the fMCAO group samples, after CV staining. The graph shows the results from all the animals evaluated by histology. **(D)** Whole brain volume measurements on the fMCAO and SHAM group samples, after IgG staining. **(E)** Oedema evaluation calculated from the IgG-stained samples ($n = 7$; $*p < 0.05$, $****p < 0.0001$, one-way ANOVA, with Tukey correction).

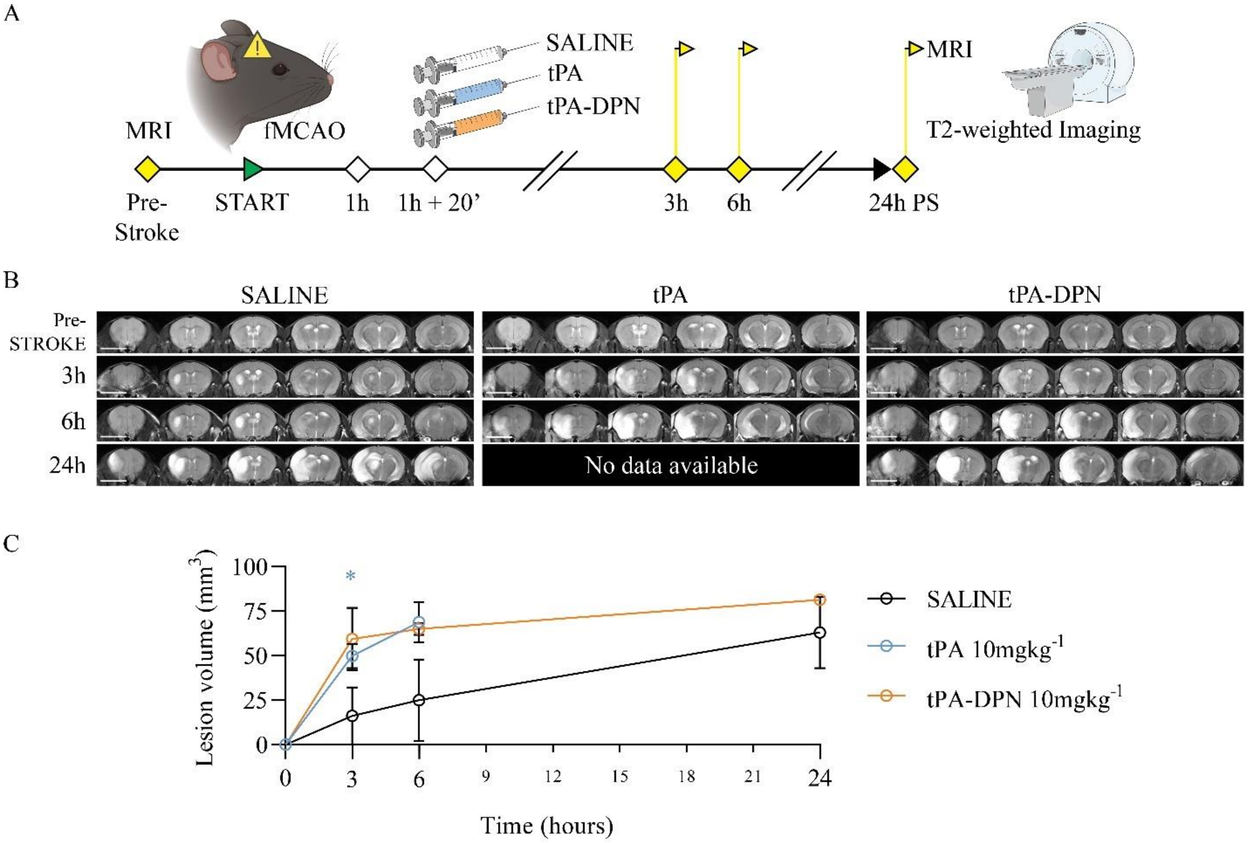

Supporting Figure 6: T_2-weighted MRI analysis for lesion size. (A) Schematic representation of the experimental plan for assessing the lesion volume via MR imaging. 24h before surgery, T_2-weighted (T_{2w}) MR imaging was performed to set the initial status of the mice. Then, MR images were taken at 3, 6 and 24h post-occlusion (fMCAO 1h) and treatment (saline, tPA 10 mg kg^{-1} or tPA-DPN 10 mg kg^{-1}). **(B)** Representative T_2-weighted images pre- and post-occlusion at different time point 3, 6, and 24h (Scale bar: 5 mm). **(C)** Quantification of the lesion volume over time. Results are expressed as mean ± SD (n=3; *$p < 0.05$; two-way ANOVA, with Tukey correction).

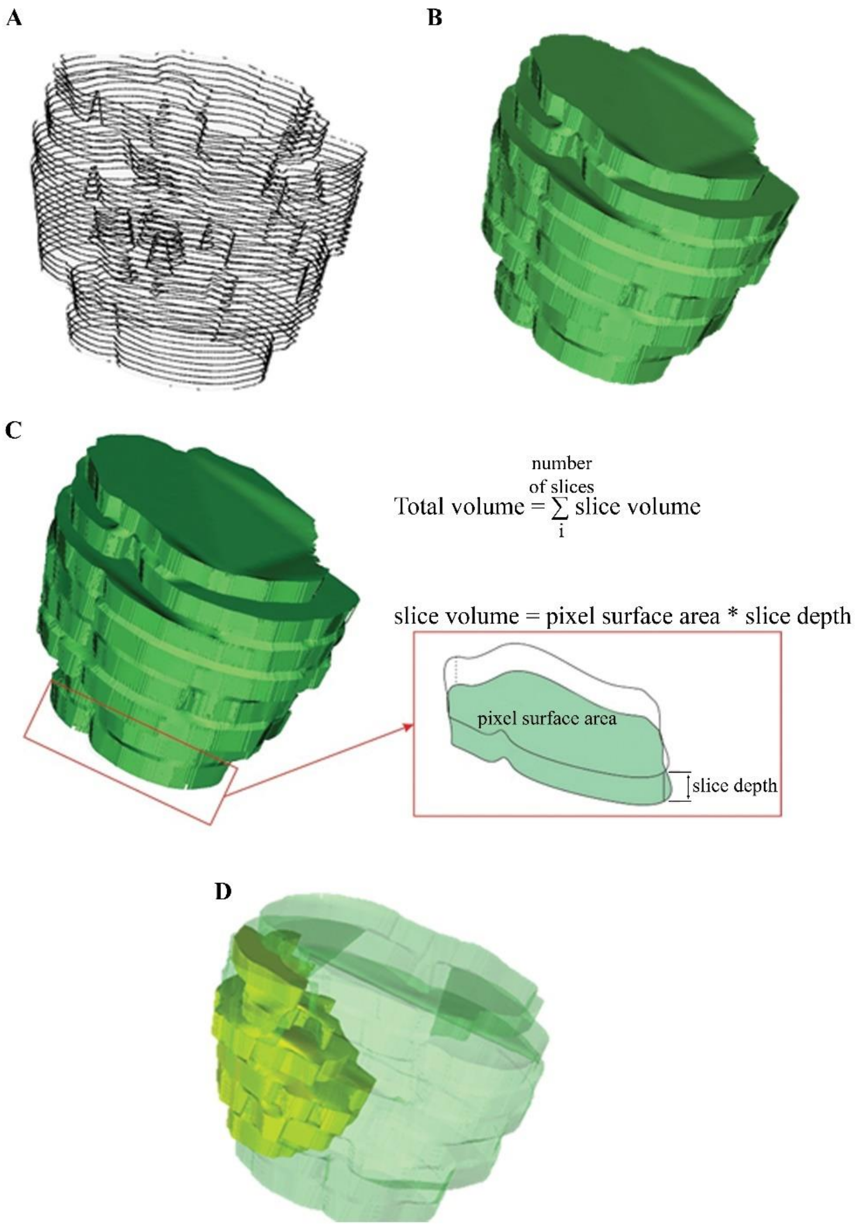

Supporting Figure 7: 3D volume reconstruction generated from histological images by an *ad-hoc* algorithm. (A) Point cloud image obtained from the algorithm. **(B)** Surface representation of the brain, obtained from the point cloud profile. **(C)** Schematic representation, and calculation aiming to obtain total volume of the reconstructed brain and lesion areas. **(D)** 3D image obtained by the overlap between the whole brain (light green) and the ischemic volume (yellow).

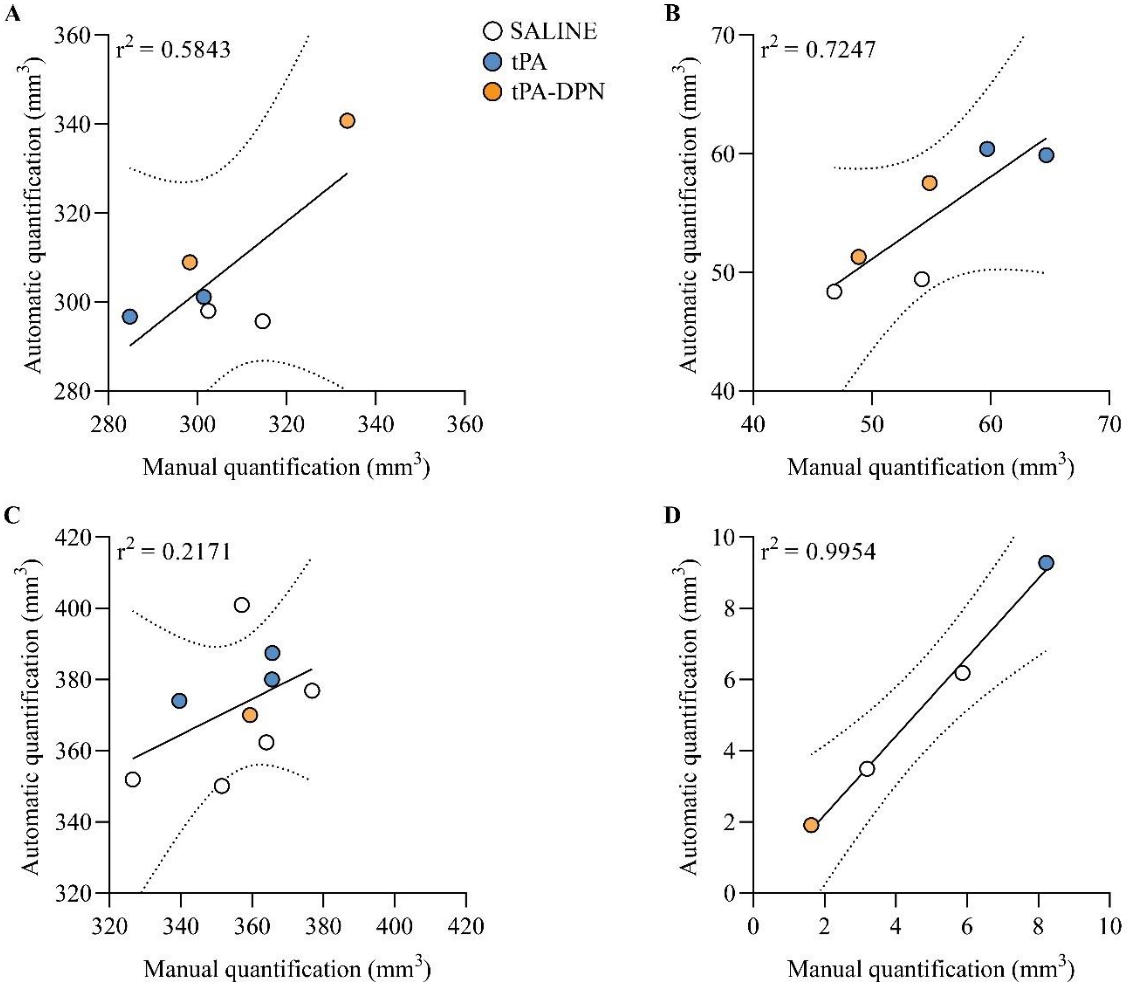

Supporting Figure 8: Comparisons between automatic quantification (from 3D reconstruction) and manual quantification. (A) Whole brain volume measurements on the fMCAO group samples, after Cresyl Violet (CV) staining. Each experimental condition is indicated by a different color, white for SALINE, light blue for free-tPA, and orange for tPA-DPN. **(B)** Lesion size measurements obtained after CV staining. **(C)** Whole brain volume measurements on the fMCAO group samples, after Immunoglobulin G (IgG) staining. **(D)** Lesion size measurements obtained after IgG staining.

***In vitro* effect of free-tPA and tPA-DPN on primary glial cells.** To further investigate the effects of free-tPA and tPA-DPN on cerebral tissues, we compared their impact in vitro on murine glial primary cells. Glial cells had a different susceptibility to tPA treatment, as revealed by IC_{50} assays (**Figure 7A – C**). In addition, astrocytes were more sensitive to tPA than microglial cells. Interestingly, the presence of a mixed population with both cell types reduced the overall sensibility to the tPA, observed as an increase of the IC_{50}. Kenna J. *et al.* evaluated the ID_{50} on primary murine astrocytes, obtaining a value around 5 μM (350 μg mL^{-1}) after 4 h exposure by metabolic [3-(4,5-dimethylthiazol-2-yl)-5-(3-carboxymethoxyphenyl)-2-(4-sulfophenyl)-2H-tetrazolium] (MTS) assay or lactate dehydrogenase (LDH) assays, and around 2 μM (140 μg mL^{-1}) by MTS and 5 μM by LDH, after 24.[1] Our results differ from those reported by Kenna et al., likely due to a higher cell density in our experiments. When we conducted an IC_{50} test using a cell density comparable to that of Kenna et al., we observed a similar IC_{50} value (unpublished data). However, under those conditions, the optical absorbance values in the MTT assay were too low, prompting us to increase the cell number to optimize the signal-to-noise ratio of the measurements. This adjustment in the protocol resulted in IC_{50} values that were an order of magnitude lower.

The co-culture model with transwell inserts was used to evaluate the efficacy of tPA-DPN in preventing the side effects of the free drug. The transwell system was specifically chosen to better simulate peripheral administration of tPA-DPN, avoiding direct contact with brain cells. Additionally, the co-culture model provides a more representative cerebral environment than single-cell-type cultures, as it incorporates glial cells responsible for immunogenic responses. Note that he transwell insert, with a pore size of 0.4 μm, simulates a compromised, hyper-permeable blood-brain barrier. In all tested conditions, the treatments (free tPA, DPN, and tPA-DN) were applied to the apical side. Free tPA was able to diffuse to the basolateral side through the insert

pores, whereas tPA conjugated to DPN remained confined to the apical side. In this setup, a dose (150 mg mL^{-1}) of tPA-DPN at 1.5 times the tPA IC_{50} for the co-culture model did not affect cell viability. In contrast, high doses (150 mg mL^{-1}) of free tPA induced toxic effects, particularly on astrocytes, causing significant cytoskeletal reorganization and cell detachment. (**Figure 8E** and **Supporting Figure 9)**.

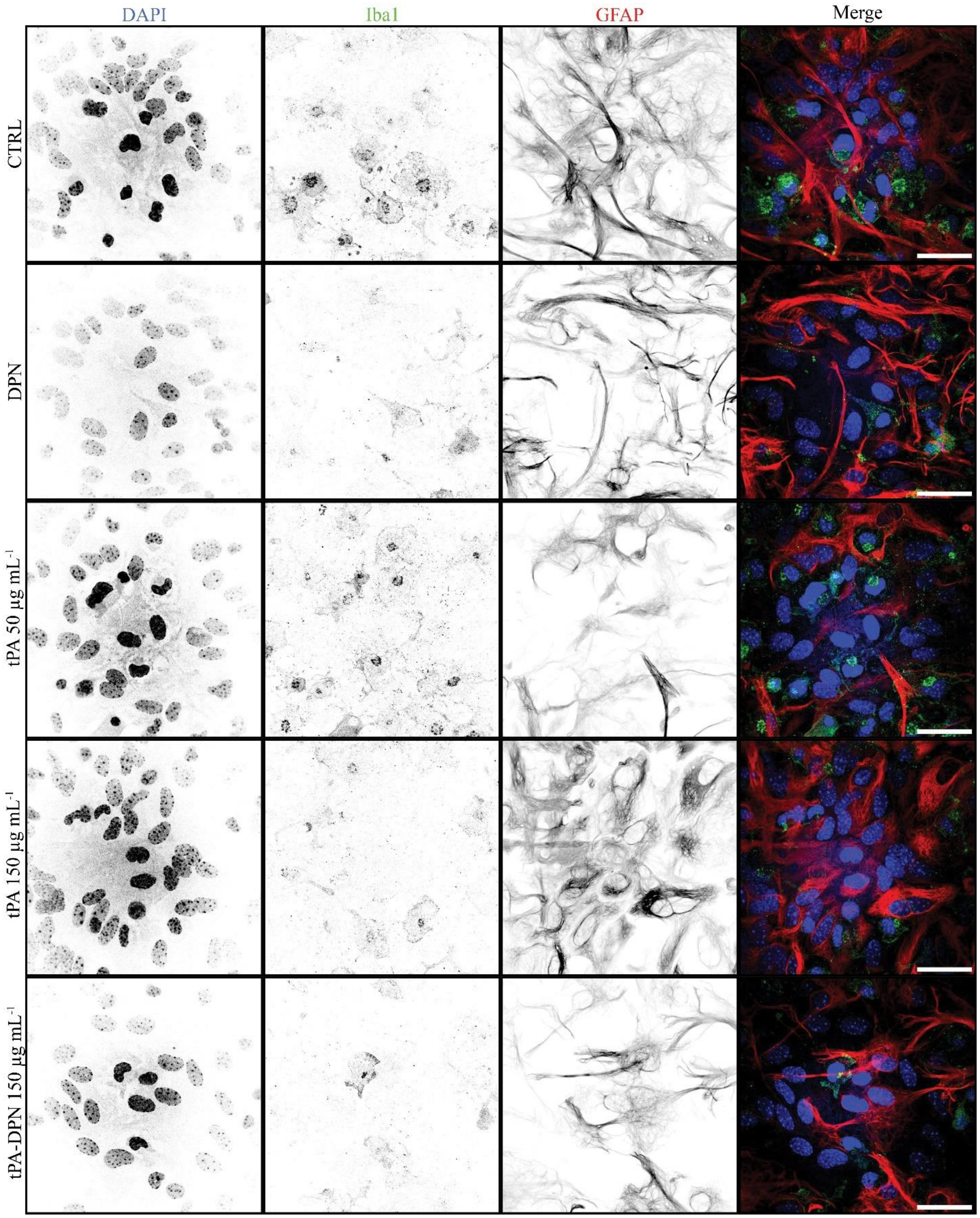

Supporting Figure 9: tPA-DPN prevents astrocyte cell morphology change in the mixed co-culture of primary murine glial cells model with Transwell insert. Immunofluorescent analysis

was conducted on the mixed co-culture of primary murine glial cells model, in presence of a porous barrier (Transwell chamber, with 0.4 µm pore-size). The different treatments (complete media, free-tPA 50 µg mL^{-1}, free-tPA 150 µg mL^{-1}, DPN and tPA-DPN 150 µg mL^{-1} were loaded inside the apical part of the insert. Representative images of the mixed co-culture stained for DAPI (nuclei, blue), Iba1 (green) and GFAP (red). Black and white images of each row of the panel show the single marker expression for each area acquired, with the last color image displaying the composite image with all the markers. The images were acquired by confocal microscopy, at 60x magnification (scale bar 50 µm).

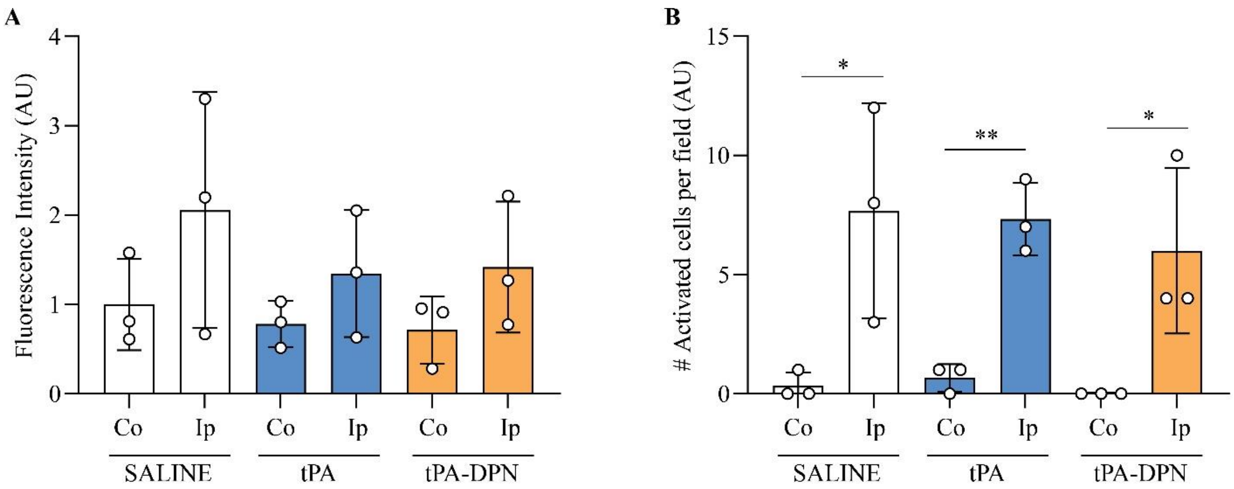

Supporting Figure 10: Immunofluorescence analysis of microglia status in stroke under the different treatments. (A) Fluorescence Intensity quantification in Co and Ip hemispheres. **(B)** Quantification of activated cells per field in Co and Ip hemispheres. Results are expressed as mean ± SD (n=3; *p < 0.05 and **p < 0.01; Two-tailed Unpaired t test).

Effect of free-tPA and tPA-DPN on primary neurons. To further investigate the effects of free-tPA and tPA-DPN on cerebral tissues, we compared their impact in vitro on murine primary neurons. These cells showed a slightly higher susceptibility to tPA treatment than the mixed glial cells population, as revealed by quantifying the characteristic lethal dose (IC_{50}). For Neurons, the IC_{50} was measured to be about 80 $\mu g \cdot mL^{-1}$, comparable with a similar quantity for the glial cells. (**Supporting Figure 11A** – neurons IC_{50} of 77.26 $\mu g \cdot mL^{-1}$ vs. mixed glial cells IC_{50} of 92.8 $\mu g \cdot mL^{-1}$). Conversely, neurons were more resistant to tPA than astrocytes (IC_{50} of 18.3 $\mu g \cdot mL^{-1}$) and less resistant than microglial cells (IC_{50} of 121.1 $\mu g \cdot mL^{-1}$). In addition, and similarly to the experiment run on the glial cells, we used the hyperpermeable BBB transwell model to test the efficacy of the tPA-DPN in preventing free-drug side effects on neurons. The transwell insert, with a pore size of 0.4 μm, simulates a compromised, hyperpermeable blood-brain barrier (**Supporting Figure 11B**). A dose of tPA-DPN (**150 $\mu g\ mL^{-1}$**), two times higher than the tPA IC_{50} for the mixed glia cells, did not affect cell viability, whereas high doses of free tPA **(150 $\mu g\ mL^{-1}$**) induced toxic effects. **Supporting Figure 11C** shows the staining of Nf200 on primary neurons exposed to empty-DPN, free tPA at two doses (50 and 150 $\mu g \cdot ml^{-1}$), and tPA-DPN with the highest tested tPA dose. Notably, the Nf200 expression for the tPA-DPN was comparable with the untreated control ($p = 0.8992$), while the free tPA led to significant neuronal damage as compared to tPA-DPN ($p = 0.0393$), returning a Nf200 expression approximately of 34% (33.61% ± 1.32%) of the control level ($p = 0.0233$).

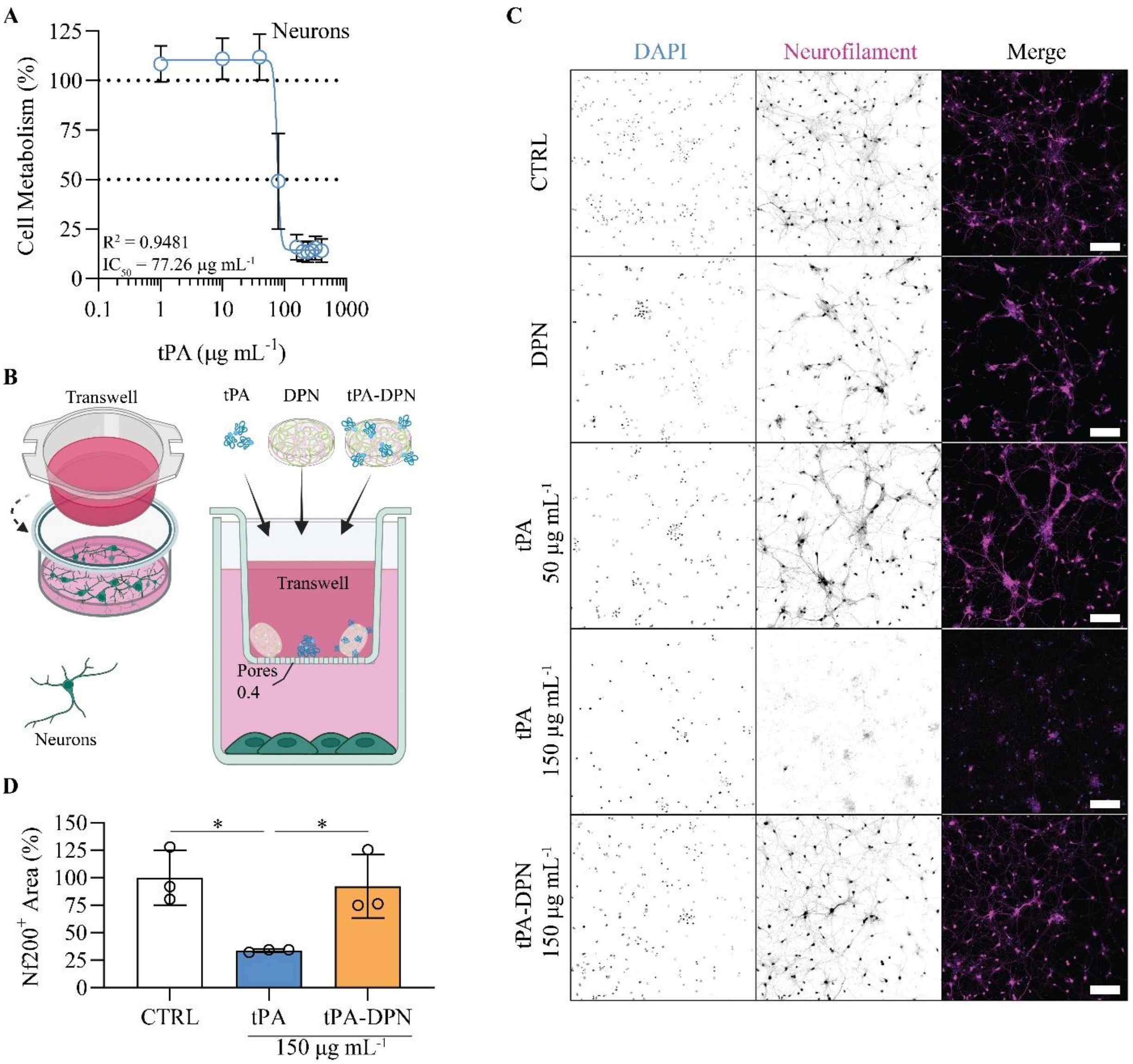

Supporting Figure 11: Effect of free-tPA and tPA-DPN on primary neurons. (A) shows the impact on cell metabolisms of 24h free-tPA treatment at different concentrations. **(B)** Schematic representation of the *in vitro* model composed by a primary culture of neuron, in presence of a porous barrier (Transwell chamber, with 0.4 µm pore-size). The different treatments (free-tPA 50 and 150 $\mu g{\cdot}mL^{-1}$, tPA-DPN 150 $\mu g{\cdot}mL^{-1}$, DPN or complete media) were loaded inside the apical part of the insert. **(C)** Representative confocal images showing cells morphology change after the different treatments. Here, nuclei were stained with DAPI (blue), while Neurofilament 200

(Nf200) was in magenta. Black and white images of each row of the panel show the single marker expression for each area acquired, with the last color image displaying the composite image with all the markers. Images were acquired at 20x magnification (scale bar: 100 μm). **(D)** Immunofluorescence analysis on Nf200 positive areas inside the region of interest, expressed as percentage. Results are expressed as mean ± SD (n = 3; $*p < 0.05$; one-way ANOVA, with Tukey Correction).

Reactive Oxygen Species (ROS) production. ROS detection was achieved by adding (2′,7′-dichlorodihydrofluorescein diacetate) ($DCFH_2$-DA) to the treatment conditions free-tPA, tPA-DPN, DPN and PBS, as from the Halo test of **Figure 2**. At 60 minutes from the beginning of the experiment, the 2′,7′-dichlorofluorescein (DCF) amount was detected to evaluate the ROS increase. Fluorescence data were normalized against the average value of the fluorescence intensity of PBS. As control for the ROS production, the $DCFH_2$-DA probe was incubated, in presence of clot or whole blood, with three different concentrations of H_2O_2 (10 pM; 10 μM and 10 mM). The **Supporting Figure 12A** shows the DCF signal fold increase for all tested conditions. Notably, only the solution with H_2O_2 returned a dose dependent increase in ROS production. Then, we performed the same experiment on whole blood, therefore in absence of thrombolysis (*i.e.* clot) **(Supporting Figure 12B)**. Once again, only the solution with H_2O_2 returned a dose dependent increase in ROS production.

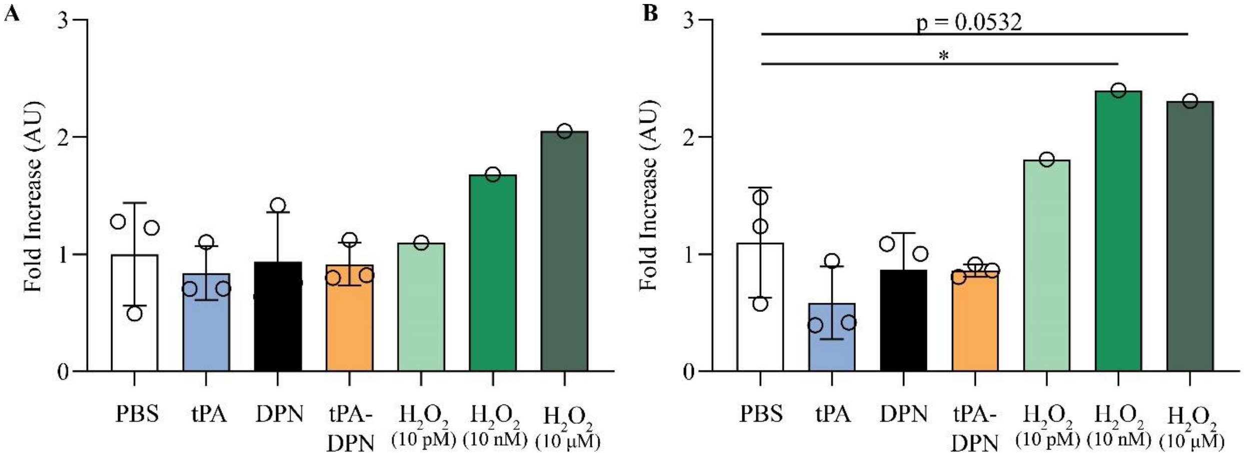

Supporting Figure 12: Reactive Oxygen Species (ROS) production during *in vitro* thrombolysis halo assay. (A) ROS fold increase 60 minutes after the start of the experiment. ROS were detected during thrombolysis by measuring the fluorescence signal associated with 2′,7′-dichlorofluorescein (DCF). **(B)** ROS fold increase 60 minutes post-incubation with whole blood (*i.e.* absence of clot). Control experiments include clot and whole blood exposed to three different concentrations of H_2O_2 (10 pM; 10 µM and 10 mM). Results are expressed as mean ± SD ($n \geq 1$; *$p < 0.05$; one-way ANOVA, with Tukey Correction).

SUPPORTING TABLES

Figure		*Type of test*	*Condition tested*	*Numerosity*	*P value*	*P value summary*
Figure 1	*E*	*Two-tailed Unpaired t test*	*tPA-DPN vs. DPN*	*≥13*	*<0.0001*	****
Figure 2	*D*	*Two-tailed Unpaired t test*	*tPA-DPN vs. DPN*	*6*	*0.1284*	*ns*
	E	*Two-tailed Unpaired t test*	*tPA-DPN vs. DPN*	*6*	*0.6648*	*ns*
	F	*Two-tailed Unpaired t test*	*tPA-DPN vs. DPN*	*6*	*0.8646*	*ns*
Figure 3	*B*	*Ordinary one-way ANOVA*	*SALINE vs. tPA*	*7*	*0.0069*	**
	B	*Ordinary one-way ANOVA*	*SALINE vs. tPA-DPN*	*7*	*0.7917*	*ns*
	B	*Ordinary one-way ANOVA*	*tPA vs. tPA-DPN*	*8*	*0.0299*	*
	C	*Ordinary one-way ANOVA*	*SALINE vs. tPA*	*>6*	*0.0079*	**
	C	*Ordinary one-way ANOVA*	*SALINE vs. tPA-DPN*	*>6*	*0.5863*	*ns*
	C	*Ordinary one-way ANOVA*	*tPA vs. tPA-DPN*	*>7*	*0.0544*	*ns*
	D	*Ordinary one-way ANOVA*	*SALINE vs. tPA*	*8*	*0.0017*	**
	D	*Ordinary one-way ANOVA*	*SALINE vs. tPA-DPN*	*>7*	*0.4283*	*ns*
	D	*Ordinary one-way ANOVA*	*tPA vs. tPA-DPN*	*>7*	*0.0394*	*
Figure 4	*B*	*Ordinary one-way ANOVA*	*SALINE vs. tPA*	*>4*	*0.0038*	**
	B	*Ordinary one-way ANOVA*	*SALINE vs. tPA-DPN*	*6*	*0.8305*	*ns*
	B	*Ordinary one-way ANOVA*	*tPA vs. tPA-DPN*	*>4*	*0.0100*	**
	E	*Ordinary one-way ANOVA*	*SALINE vs. tPA*	*6*	*0.0020*	**
	E	*Ordinary one-way ANOVA*	*SALINE vs. tPA-DPN*	*4*	*0.8847*	*ns*
	E	*Ordinary one-way ANOVA*	*tPA vs. tPA-DPN*	*6*	*0.0043*	**
Figure 5	*B*	*Ordinary one-way ANOVA*	*SALINE vs. tPA*	*7*	*0.0323*	*
	B	*Ordinary one-way ANOVA*	*SALINE vs. tPA-DPN*	*7*	*0.9952*	*ns*
	B	*Ordinary one-way ANOVA*	*tPA vs. tPA-DPN*	*7*	*0.0390*	*
	E	*Ordinary one-way ANOVA*	*SALINE vs. tPA*	*>5*	*0.1044*	*ns*
	E	*Ordinary one-way ANOVA*	*SALINE vs. tPA-DPN*	*>5*	*0.8120*	*ns*
	E	*Ordinary one-way ANOVA*	*tPA vs. tPA-DPN*	*6*	*0.0260*	*
Figure 6	*C*	*Ordinary one-way ANOVA*	*CTRL vs. tPA*	*3*	*0.0021*	**
	C	*Ordinary one-way ANOVA*	*CTRL vs. tPA-DPN*	*3*	*0.0650*	*ns*
	C	*Ordinary one-way ANOVA*	*tPA vs. tPA-DPN*	*3*	*0.0003*	***
Figure 7	*F*	*Ordinary one-way ANOVA*	*CTRL vs. tPA*	*3*	*0.6875*	*ns*
	F	*Ordinary one-way ANOVA*	*CTRL vs. tPA-DPN*	*3*	*0.3296*	*ns*
	F	*Ordinary one-way ANOVA*	*tPA vs. tPA-DPN*	*3*	*0.7653*	*ns*
	G	*Ordinary one-way ANOVA*	*CTRL vs. tPA*	*3*	*0.0437*	*
	G	*Ordinary one-way ANOVA*	*CTRL vs. tPA-DPN*	*3*	*0.9101*	*ns*
	G	*Ordinary one-way ANOVA*	*tPA vs. tPA-DPN*	*3*	*0.0733*	*ns*

Figure 8	*B*	*Two-tailed Unpaired t test*	*SALINE Co vs. SALINE Ip*	*5*	*0.3514*	*ns*
	B	*Two-tailed Unpaired t test*	*SALINE Co vs. tPA Co*	*>3*	*0.6064*	*ns*
	B	*Two-tailed Unpaired t test*	*SALINE Co vs. tPA Ip*	*>3*	*0.9850*	*ns*
	B	*Two-tailed Unpaired t test*	*SALINE Co vs. tPA-DPN Co*	*>4*	*0.9845*	*ns*
	B	*Two-tailed Unpaired t test*	*SALINE Co vs. tPA-DPN Ip*	*>4*	*0.6960*	*ns*
	B	*Two-tailed Unpaired t test*	*SALINE Ip vs. tPA Co*	*>3*	*0.2382*	*ns*
	B	*Two-tailed Unpaired t test*	*SALINE Ip vs. tPA Ip*	*>3*	*0.4243*	*ns*
	B	*Two-tailed Unpaired t test*	*SALINE Ip vs. tPA-DPN Co*	*>4*	*0.3355*	*ns*
	B	*Two-tailed Unpaired t test*	*SALINE Ip vs. tPA-DPN Ip*	*>4*	*0.8227*	*ns*
	D	*Two-tailed Unpaired t test*	*SALINE Ip vs. SALINE Co.*	*5*	*0.0183*	** (alpha 0.1)*
	D	*Two-tailed Unpaired t test*	*tPA Ip vs. tPA Co.*	*3*	*0.3657*	*ns*
	D	*Two-tailed Unpaired t test*	*tPA-DPN Ip vs. tPA-DPN Co*	*4*	*0.0658*	** (alpha 0.1)*
	D	*Two-tailed Unpaired t test*	*tPA Co vs. SALINE Co.*	*>3*	*0.5340*	*ns*
	D	*Two-tailed Unpaired t test*	*tPA-DPN Co vs. SALINE Co*	*>4*	*0.5410*	*ns*
	D	*Two-tailed Unpaired t test*	*tPA-DPN Co vs. tPA Co*	*>3*	*0.2487*	*ns*
	D	*Two-tailed Unpaired t test*	*tPA Ip vs. SALINE Ip*	*>3*	*0.1232*	*ns*
	D	*Two-tailed Unpaired t test*	*tPA-DPN Ip vs. SALINE Ip*	*>4*	*0.5048*	*ns*
	D	*Two-tailed Unpaired t test*	*tPA-DPN Ip vs. tPA Ip*	*>3*	*0.2512*	*ns*
Supplementary Figures		***Type of test***	***Condition tested***	***Numerosity***	***P value***	***P value summary***
Supporting Figure 1	*A*	*Log-rank (Mantel-Cox) test*	*Whole*	*≥7*	*0.0019*	****
	A	*Log-rank (Mantel-Cox) test*	*SALINE vs. tPA*	*8*	*0.0016*	****
	A	*Log-rank (Mantel-Cox) test*	*SALINE vs. tPA-DPN*	*≥7*	*0.3995*	*ns*
	A	*Log-rank (Mantel-Cox) test*	*tPA vs. tPA-DPN*	*≥7*	*0.0143*	***
	C	*Ordinary one-way ANOVA*	*SHAM SALINE vs. SHAM tPA*	*>4*	*0.9998*	*ns*
	C	*Ordinary one-way ANOVA*	*SHAM SALINE vs. SHAM tPA-DPN*	*4*	*>0.9999*	*ns*
	C	*Ordinary one-way ANOVA*	*SHAM SALINE vs. fMCAO SALINE*	*>4*	*0.2166*	*ns*
	C	*Ordinary one-way ANOVA*	*SHAM SALINE vs. fMCAO tPA*	*>4*	*<0.0001*	******
	C	*Ordinary one-way ANOVA*	*SHAM SALINE vs. fMCAO tPA-DPN*	*>4*	*0.0270*	***
	C	*Ordinary one-way ANOVA*	*SHAM tPA vs. SHAM tPA-DPN*	*>4*	*>0.9999*	*ns*
	C	*Ordinary one-way ANOVA*	*SHAM tPA vs. fMCAO SALINE*	*6*	*0.0724*	*ns*
	C	*Ordinary one-way ANOVA*	*SHAM tPA vs. fMCAO tPA*	*>6*	*<0.0001*	******
	C	*Ordinary one-way ANOVA*	*SHAM tPA vs. fMCAO tPA-DPN*	*>6*	*0.0046*	****
	C	*Ordinary one-way ANOVA*	*SHAM tPA-DPN vs. fMCAO SALINE*	*>4*	*0.2014*	*ns*
	C	*Ordinary one-way ANOVA*	*SHAM tPA-DPN vs. fMCAO tPA*	*>4*	*<0.0001*	******
	C	*Ordinary one-way ANOVA*	*SHAM tPA-DPN vs. fMCAO tPA-DPN*	*>4*	*0.0244*	***
	C	*Ordinary one-way ANOVA*	*fMCAO SALINE vs. fMCAO tPA*	*>6*	*0.0148*	***
	C	*Ordinary one-way ANOVA*	*fMCAO SALINE vs. fMCAO tPA-DPN*	*>6*	*0.9014*	*ns*
	C	*Ordinary one-way ANOVA*	*fMCAO tPA vs. fMCAO tPA-DPN*	*>7*	*0.1289*	*ns*
	D	*Ordinary one-way ANOVA*	*SHAM SALINE vs. SHAM tPA*	*6*	*>0.9999*	*ns*
	D	*Ordinary one-way ANOVA*	*SHAM SALINE vs. SHAM tPA-DPN*	*6*	*>0.9999*	*ns*
	D	*Ordinary one-way ANOVA*	*SHAM SALINE vs. fMCAO SALINE*	*>6*	*0.0254*	***
	D	*Ordinary one-way ANOVA*	*SHAM SALINE vs. fMCAO tPA*	*>6*	*<0.0001*	******
	D	*Ordinary one-way ANOVA*	*SHAM SALINE vs. fMCAO tPA-DPN*	*>6*	*0.0005*	*****

Supporting Figure 1	*D*	*Ordinary one-way ANOVA*	*SHAM tPA vs. SHAM tPA-DPN*	*>6*	*>0.9999*	*ns*
	D	*Ordinary one-way ANOVA*	*SHAM tPA vs. fMCAO SALINE*	*6*	*0.0254*	*
	D	*Ordinary one-way ANOVA*	*SHAM tPA vs. fMCAO tPA*	*>6*	*<0.0001*	****
	D	*Ordinary one-way ANOVA*	*SHAM tPA vs. fMCAO tPA-DPN*	*>6*	*0.0005*	***
	D	*Ordinary one-way ANOVA*	*SHAM tPA-DPN vs. fMCAO SALINE*	*>6*	*0.0254*	*
	D	*Ordinary one-way ANOVA*	*SHAM tPA-DPN vs. fMCAO tPA*	*>6*	*<0.0001*	****
	D	*Ordinary one-way ANOVA*	*SHAM tPA-DPN vs. fMCAO tPA-DPN*	*>6*	*0.0005*	***
	D	*Ordinary one-way ANOVA*	*fMCAO SALINE vs. fMCAO tPA*	*8*	*0.0001*	***
	D	*Ordinary one-way ANOVA*	*fMCAO SALINE vs. fMCAO tPA-DPN*	*>7*	*0.5791*	*ns*
	D	*Ordinary one-way ANOVA*	*fMCAO tPA vs. fMCAO tPA-DPN*	*>7*	*0.0185*	*
Supporting Figure 2		*Two-tailed Unpaired t test*	*tPA-DPN vs. DPN*	*6*	*0.0500*	*
Supporting Figure 5	*A*	*Ordinary one-way ANOVA*	*SHAM SALINE vs. SHAM tPa*	*>3*	*0.1326*	*ns*
	A	*Ordinary one-way ANOVA*	*SHAM SALINE vs. SHAM tPA-DPN*	*>3*	*0.6507*	*ns*
	A	*Ordinary one-way ANOVA*	*SHAM SALINE vs. fMCAO SALINE*	*>3*	*0.1249*	*ns*
	A	*Ordinary one-way ANOVA*	*SHAM SALINE vs. fMCAO tPA*	*7*	*0.5171*	*ns*
	A	*Ordinary one-way ANOVA*	*SHAM SALINE vs. fMCAO tPA-DPN*	*7*	*0.2115*	*ns*
	A	*Ordinary one-way ANOVA*	*SHAM tPA vs. SHAM tPA-DPN*	*>3*	*0.8825*	*ns*
	A	*Ordinary one-way ANOVA*	*SHAM tPA vs. fMCAO SALINE*	*>3*	*0.9974*	*ns*
	A	*Ordinary one-way ANOVA*	*SHAM tPA vs. fMCAO tPA*	*>3*	*0.7571*	*ns*
	A	*Ordinary one-way ANOVA*	*SHAM tPA vs. fMCAO tPA-DPN*	*7*	*0.9754*	*ns*
	A	*Ordinary one-way ANOVA*	*SHAM tPA-DPN vs. fMCAO SALINE*	*7*	*0.9575*	*ns*
	A	*Ordinary one-way ANOVA*	*SHAM tPA-DPN vs. fMCAO tPA*	*>3*	*>0.9999*	*ns*
	A	*Ordinary one-way ANOVA*	*SHAM tPA-DPN vs. fMCAO tPA-DPN*	*>3*	*0.9934*	*ns*
	A	*Ordinary one-way ANOVA*	*fMCAO SALINE vs. fMCAO tPA*	*>3*	*0.8509*	*ns*
	A	*Ordinary one-way ANOVA*	*fMCAO SALINE vs. fMCAO tPA-DPN*	*7*	*0.9989*	*ns*
	A	*Ordinary one-way ANOVA*	*fMCAO tPA vs. fMCAO tPA-DPN*	*7*	*0.9669*	*ns*
	B	*Two-tailed Unpaired t test*	*fMCAO vs. SHAM*	*>3*	*<0.0001*	****
	C	*Ordinary one-way ANOVA*	*SALINE vs. tPA*	*7*	*0.0644*	* *(alpha 0.1)*
	C	*Ordinary one-way ANOVA*	*SALINE vs. tPA-DPN*	*7*	*0.9801*	*ns*
	C	*Ordinary one-way ANOVA*	*tPA vs. tPA-DPN*	*7*	*0.0926*	* *(alpha 0.1)*
	D	*Ordinary one-way ANOVA*	*SHAM SALINE vs. SHAM tPa*	*>3*	*0.2224*	*ns*
	D	*Ordinary one-way ANOVA*	*SHAM SALINE vs. SHAM tPA-DPN*	*>3*	*0.8769*	*ns*
	D	*Ordinary one-way ANOVA*	*SHAM SALINE vs. fMCAO SALINE*	*>3*	*0.4478*	*ns*
	D	*Ordinary one-way ANOVA*	*SHAM SALINE vs. fMCAO tPA*	*7*	*0.7095*	*ns*
	D	*Ordinary one-way ANOVA*	*SHAM SALINE vs. fMCAO tPA-DPN*	*7*	*0.6459*	*ns*
	D	*Ordinary one-way ANOVA*	*SHAM tPA vs. SHAM tPA-DPN*	*>3*	*0.7559*	*ns*
	D	*Ordinary one-way ANOVA*	*SHAM tPA vs. fMCAO SALINE*	*>3*	*0.8959*	*ns*
	D	*Ordinary one-way ANOVA*	*SHAM tPA vs. fMCAO tPA*	*>3*	*0.7132*	*ns*
	D	*Ordinary one-way ANOVA*	*SHAM tPA vs. fMCAO tPA-DPN*	*7*	*0.7649*	*ns*
	D	*Ordinary one-way ANOVA*	*SHAM tPA-DPN vs. fMCAO SALINE*	*7*	*0.9925*	*ns*
	D	*Ordinary one-way ANOVA*	*SHAM tPA-DPN vs. fMCAO tPA*	*>3*	*>0.9999*	*ns*

Supporting Figure 5	*D*	*Ordinary one-way ANOVA*	*SHAM tPA-DPN vs. fMCAO tPA-DPN*	*>3*	*0.9999*	*ns*
	D	*Ordinary one-way ANOVA*	*fMCAO SALINE vs. fMCAO tPA*	*>3*	*0.9927*	*ns*
	D	*Ordinary one-way ANOVA*	*fMCAO SALINE vs. fMCAO tPA-DPN*	*7*	*0.9980*	*ns*
	D	*Ordinary one-way ANOVA*	*fMCAO tPA vs. fMCAO tPA-DPN*	*7*	*>0.9999*	*ns*
	E	*Two-tailed Unpaired t test*	*fMCAO vs. SHAM*	*>3*	*<0.0001*	****
Supporting Figure 6	*C*	*Mixed-effects analysis*	*SALINE 3h vs. tPA 3h*	*>3*	*0.0381*	*
	C	*Mixed-effects analysis*	*SALINE 3h vs. tPA-DPN 3h*	*>3*	*0.0571*	*ns*
	C	*Mixed-effects analysis*	*tPA 3h vs. tPA-DPN 3h*	*3*	*0.6938*	*ns*
	C	*Mixed-effects analysis*	*SALINE 6h vs. tPA 6h*	*>3*	*0.0522*	*ns*
	C	*Mixed-effects analysis*	*SALINE 6h vs. tPA-DPN 6h*	*>3*	*0.0736*	*ns*
	C	*Mixed-effects analysis*	*tPA 6h vs. tPA-DPN 6h*	*3*	*0.8652*	*ns*
Supporting Figure 10	*A*	*Two-tailed Unpaired t test*	*SALINE Ip vs. SALINE Co.*	*3*	*0.2663*	*ns*
	A	*Two-tailed Unpaired t test*	*tPA Ip vs. tPA Co.*	*3*	*0.2657*	*ns*
	A	*Two-tailed Unpaired t test*	*tPA-DPN Ip vs. tPA-DPN Co*	*3*	*0.2133*	*ns*
	A	*Two-tailed Unpaired t test*	*tPA Co vs. SALINE Co.*	*3*	*0.5473*	*ns*
	A	*Two-tailed Unpaired t test*	*tPA-DPN Co vs. SALINE Co*	*3*	*0.4812*	*ns*
	A	*Two-tailed Unpaired t test*	*tPA-DPN Co vs. tPA Co*	*3*	*0.8116*	*ns*
	A	*Two-tailed Unpaired t test*	*tPA Ip vs. SALINE Ip*	*3*	*0.4590*	*ns*
	A	*Two-tailed Unpaired t test*	*tPA-DPN Ip vs. SALINE Ip*	*3*	*0.5059*	*ns*
	A	*Two-tailed Unpaired t test*	*tPA-DPN Ip vs. tPA Ip*	*3*	*0.9083*	*ns*
	B	*Two-tailed Unpaired t test*	*SALINE Ip vs. SALINE Co.*	*3*	*0.0491*	*
	B	*Two-tailed Unpaired t test*	*tPA Ip vs. tPA Co.*	*3*	*0.0021*	**
	B	*Two-tailed Unpaired t test*	*tPA-DPN Ip vs. tPA-DPN Co*	*3*	*0.0399*	*
	B	*Two-tailed Unpaired t test*	*tPA Co vs. SALINE Co.*	*3*	*0.5185*	*ns*
	B	*Two-tailed Unpaired t test*	*tPA-DPN Co vs. SALINE Co*	*3*	*0.3739*	*ns*
	B	*Two-tailed Unpaired t test*	*tPA-DPN Co vs. tPA Co*	*3*	*0.1161*	*ns*
	B	*Two-tailed Unpaired t test*	*tPA Ip vs. SALINE Ip*	*3*	*0.9093*	*ns*
	B	*Two-tailed Unpaired t test*	*tPA-DPN Ip vs. SALINE Ip*	*3*	*0.6384*	*ns*
	B	*Two-tailed Unpaired t test*	*tPA-DPN Ip vs. tPA Ip*	*3*	*0.5748*	*ns*
Supporting Figure 11	*D*	*Ordinary one-way ANOVA*	*CTRL vs. tPA*	*3*	*0.0233*	*
	D	*Ordinary one-way ANOVA*	*CTRL vs. tPA-DPN*	*3*	*0.8992*	*ns*
	D	*Ordinary one-way ANOVA*	*tPA vs. tPA-DPN*	*3*	*0.0393*	*
Supporting Figure 12	*A*	*Ordinary one-way ANOVA*	*PBS vs.* H_2O_2 *10 µM*	*≥1*	*0.0532*	*ns*
	A	*Ordinary one-way ANOVA*	*PBS vs.* H_2O_2 *10 nM*	*≥1*	*0.0375*	*
	A	*Ordinary one-way ANOVA*	*PBS vs.* H_2O_2 *10 pM*	*≥1*	*0.3430*	*ns*
	A	*Ordinary one-way ANOVA*	*PBS vs. tPA*	*3*	*0.3232*	*ns*
	A	*Ordinary one-way ANOVA*	*PBS vs. DPN*	*3*	*0.8999*	*ns*
	A	*Ordinary one-way ANOVA*	*PBS vs. tPA-DPN*	*3*	*0.8890*	*ns*

Supporting Figure 12	*B*	*Ordinary one-way ANOVA*	*PBS vs.* H_2O_2 *10 μM*	*≥1*	*0.1151*	*ns*
	B	*Ordinary one-way ANOVA*	*PBS vs.* H_2O_2 *10 nM*	*≥1*	*0.4129*	*ns*
	B	*Ordinary one-way ANOVA*	*PBS vs.* H_2O_2 *10 pM*	*≥1*	*0.9998*	*ns*
	B	*Ordinary one-way ANOVA*	*PBS vs. tPA*	*3*	*0.9999*	*ns*
	B	*Ordinary one-way ANOVA*	*PBS vs. DPN*	*3*	*0.9822*	*ns*
	B	*Ordinary one-way ANOVA*	*PBS vs. tPA-DPN*	*3*	*0.9994*	*ns*

Supporting Table 1. Statistical Analyses. List of all the statistical analyses presented in the manuscript, with details on the methodsnumerosity and calculated p values.

A

Type and Sample number	Manual Measure (mm^3)	Automatic Measure (mm^3)	Diff. (mm^3)
Brain 1	314.6	295.72	18.88
Brain 2	284.83	296.78	-11.95
Brain 3	302.46	298.02	4.44
Brain 4	298.32	308.98	-10.66
Brain 5	301.43	301.14	0.29
Brain 6	333.63	340.74	-7.11
Lesion 1	54.21	49.43	4.78
Lesion 2	64.69	59.9	4.79
Lesion 3	46.84	48.39	-1.55
Lesion 4	54.87	57.55	-2.68
Lesion 5	59.7	60.42	-0.72
Lesion 6	48.89	51.31	-2.42

B

Type and Sample number	Manual Measure (mm^3)	Automatic Measure (mm^3)	Diff. (mm^3)
Brain 1	364.01	362.41	1.60
Brain 2	351.52	350.12	1.40
Brain 3	376.88	376.87	0.01
Brain 4	326.55	352	-25.45
Brain 5	357.1	400.97	-43.87
Brain 6	365.6	380	-14.40
Brain 7	339.66	374.07	-34.41
Brain 8	365.71	387.42	-21.71
Brain 9	359.46	370.03	-10.57
Lesion 1	5.87	6.19	-0.32
Lesion 2	3.2	3.5	-0.30
Lesion 3	43.97	53.38	-9.41
Lesion 4	8.22	9.28	-1.06
Lesion 5	1.63	1.91	-0.28

Supporting Table 2. Summary and comparison of the data obtained from the manual and automatic measurements on the histological samples. The whole brain and lesion volume results obtained from histological sections (**A**) or MR images (**B**) were compared to the data calculated automatically after the 3D reconstruction shown in **Supporting Figure 7.** The parameter 'Difference' (Diff.) displays the divergence between these Manual and Automatic

Measures. A positive deviation between the two numbers is highlighted in green color, while a negative value is in red color.

SUPPORTING METHODS

Filament middle cerebral artery occlusion (fMCAO) stroke model. Surgical tools (Product codes 15000-08, 11253-29, 11700-03, 11274-20, 14060-09, 14058-09 and 91201-13), and Nonabsorbable 5-0 Braided Silk Suture (No. 18020-50) were purchased by Fine Science Tools (FST) company (Fine Science Tools GmbH, Heidelberg, DE).

3D volume reconstruction generated from histological images by an ad-hoc algorithm. To demonstrate a possible spatial co-localization between the lesion and the zone with enhanced vascular permeability (IgG extravasation), we performed a 3D volume reconstruction from the histological sections using an *ad hoc* algorithm (**Supporting Figure 7**). The 3D model was tuned and validated on the results obtained from the histological analyses (**Supporting Figure 8**). Data obtained from the manual measurements on the histological sections were compared to the data calculated automatically after the 3D reconstruction, confirming the accuracy of the method (**Supporting Table 2**). The 3D reconstructions (**Figure 4E**) demonstrated a good overlapping between the damaged tissue (lesion) and the vascular hyper-permeable zones for all three treatment groups.

Algorithm for 3D reconstruction

```
createPointCloud
        # creating a point cloud file (.ply) from numpy array
```

addPoints

    # extracting points from mask and adds to list

main

    # defining parameters for conversion

- pixel to mm conversion
- distance between slices
- kernel for erosion/dilatation

    # loading the 8-bit tiff mask images

    # performing erosion on odd images creating an additional image

    # performing dilatation on even images creating an additional image

    # stacking all images with defined distance between slices

    # detecting edges on all newly created images using Canny edge detection

    # converting images to masks

    # getting contour points and adding to the list using function addPoints

- saving to point cloud file using function createPointCloud

Primary rat cortical neurons preparation.

HBSS #14170088; Neurobasal NB, #21103049; GlutaMAX™, #35050038; B-27™, #17504044; and HS, #26050088; were purchased from Gibco™, Thermo Fisher Scientific, MA, USA. Trypsin, #25050014; DNase I, #D5025; P/S, #P4333; and PDL, #P6407; were purchased from Sigma-Aldrich®, Merck KGaA, Germany. Plates were purchased from Corning®, NY, USA.

Primary cortical neurons were prepared from rat embryos at embryonic day E17 (pregnant rats from Charles River Laboratories, Italy). After the pregnant rats were sacrificed, all the steps were performed in Petri dishes with ice-cold HBSS and sitting on ice. The embryos were extracted from the placenta and decapitated with scissors. The brains were extracted, the two hemispheres were divided, and the cortices were obtained by discarding the meninges, the corpus striatum and the hippocampus. Cortical tissue was digested for 30 min at 37°C in 0.125% Trypsin/HBSS with 25 μg DNase I per mL of solution (1.3 mL of digestion solution per cortex). Digestion was stopped by adding complete Neurobasal medium (NB), supplemented with 1% P/S, 1% GlutaMAX™, 2% B-27™ and 10% heat-inactivated horse serum (HS). After 5 min centrifugation at 275 rcf, the supernatant was discarded, and the tissue was dissociated through gentle pipetting in freshly added 10% HS NB. Any remaining tissue fragments were filtered out through a 40 μm strainer. After 7 min centrifugation at 94 rcf, cells were resuspended in complete NB and plated at the desired density on 0.01% PDL-coated surfaces (Poly-D-lysine/ddH2O). Cells were grown at 37 °C in a humid atmosphere of 5% CO2. Half of the cell culture media was replaced with fresh complete NB every 3-7 days.

Cell Metabolism analysis. To perform MTT, Neurons were cultured in 96-well plates, at a cell density of 170,000 cells/cm^2 (50,000 cells/well), and maintained at 37 °C in 5% CO2, in NB, supplemented with 1% P/S, 1% GlutaMAX™, 2% B-27™ and 10% heat-inactivated HS. Cells were treated with different concentrations of free-tPA (namely, 1, 10, 40, 80, 160, 200, 240, 280, 320, 400 μg mL^{-1}), or an equivalent number of empty DPN matching the different tPA-DPN concentrations. At the end of the designated incubation times, 5 mg mL^{-1} of MTT solution in DPBS was added to each well, and the cells were incubated for 4 h at 37 °C. The solubilized formazan

product was dissolved with absolute alcohol (100-200 µL/well) and quantified using a spectrophotometer at 570 nm, using 650 nm as the reference wavelength (Tecan, Männedorf, Swiss). The percentage of cell metabolism was assessed according to the equation (3) described in the main text.

Hyperpermeable BBB transwell experiments with primary rat cortical neurons. Primary rat cortical neurons were prepared as described above, seeded in 24-well plates at 35,000/cm^2 (75,000 cells/well) in complete NB media and cultured at 37°C in humidified 5% CO_2 for 7 days. Cells were treated for 24 h with different concentrations of free-tPA (150 µg mL^{-1}), tPA-DPN (150 µg mL^{-1}), tPA-DPN (50 µg mL^{-1}) and corresponding amount of DPN. Cell morphology, immunofluorescence analysis with signal intensity quantification were performed.

Immunofluorescence on primary neurons. Neurons were both cultured on glass coverslips in 24-well plates at a cell density of 35,000/cm^2 (75,000 cells/well) with 12Ø glass coverslips before fixation with 4% PFA for 15 min at RT. To perform the staining, cells were then permeabilized for 15 min with 0.7% Triton™ X-100, prior to 2× washing steps with PBS and 30 min blocking with 1% BSA in DPBS. Room temperature incubation with antibodies followed for 1 hour each (primary rabbit anti-neurofilament heavy NF200 antibody, secondary goat anti-rabbit IgG AF647 antibody, 1:800 dilution v/v in 1% BSA in DPBS). After each incubation with antibodies, cells were washed 2× for 5 min with PBS for and 2× for 10 min with 0.01% Tween® 20. All dilutions, except antibodies, were done in PBS. After 10 min incubation with 10 µg mL^{-1} DAPI at RT and dipping in ddH2O, cells were mounted with ProLong™ Diamond Antifade Mountant.

***In vitro* Reactive Oxygen Species (ROS) production over halo clot thrombolytic assay.** To evaluate the ROS production over thrombolysis, we performed the *in vitro* halo clot assay in presence of $DCFH_2$-DA (2’,7’-Dichlorodihydrofluorescin diacetate) probe (Merck Sigma-Aldrich, product code D6883-50MG-PW). $DCFH_2$-DA probe is deacetylated by esterases to DCFH (2′,7′-Dichlorodihydrofluorescin), which in the presence of oxidants (*i.e.* ROS), is converted into DCF (2′,7′-Dichlorofluorescin). $DCFH_2$-DA probe was dissolved into DMSO at the concentration of 10 mM (stock solution) to finally use at the concentration of 10 μM (working solution). 10 μM $DCFH_2$-DA was added to the treatment condition, right before the addition to each well. As positive controls of ROS formation, a series of wells were prepared adding H_2O_2 (as treatment condition), diluted in DPBS1x at the concentration of 10 pM, 10 nM and 10 μM. Free-tPA or tPA-DPN were diluted into saline solution and 3 μg of each treatment was added in each well (final volume considering a clot volume equal to 200 μL, concentration of 214.3 nM). Each condition was tested at least in technical quadruplicate. The test was performed for 1 h, at 37°C. Right after the addition of all the treatments, clot degradation was followed by the absorbance change at 510 nm every 2 min, using a Tecan Spark® (Tecan, Männedorf, CH). Additionally, DCF fluorescence was detected every 2 min, through the excitation and emission wavelengths of 485 nm and 530 nm, respectively.

SUPPORTING REFERENCES

(1) Kenna, J. E.; Anderton, R. S.; Knuckey, N. W.; Meloni, B. P. Assessment of recombinant tissue plasminogen activator (rtPA) toxicity in cultured neural cells and subsequent treatment with poly-arginine peptide R18D. *Neurochem Res* **2020**, *45* (5), 1215-1229. DOI: 10.1007/s11064-020-03004-3 From NLM Medline.
